# The Interaction Effect of the Design Parameters on the Water Absorption of the Hemp-Reinforced Biocarbon-Filled Bio-Epoxy Composites

**DOI:** 10.3390/ijms24076093

**Published:** 2023-03-23

**Authors:** Raj Kumar Dahal, Bishnu Acharya, Animesh Dutta

**Affiliations:** 1 Bio-Renewable Innovation Lab, School of Engineering, University of Guelph, Guelph, ON N1G 2W1, Canada; 2 Department of Chemical and Biological Engineering, University of Saskatchewan, Saskatoon, SK S7N 5A9, Canada

**Keywords:** biocarbon, biofillers, hemp composites, sustainable material, water absorption, green economy

## Abstract

Natural fiber-reinforced composites perform poorly when exposed to moisture. Biocarbon has been proven to improve the water-absorbing behavior of natural fiber composites. However, the interaction effect of the design parameters on the biocarbon-filled hemp fiber-reinforced bio-epoxy composites has not been studied. In this study, the effects of the design parameters (pyrolysis temperature, biocarbon particle size, and filler loading) on the water absorptivity and water diffusivity of hemp-reinforced biopolymer composites have been investigated. Biocarbon from the pyrolysis of hemp and switchgrass was produced at 450, 550, and 650 °C. Composite samples with 10 wt.%, 15 wt.%, and 20 wt.% of biocarbon fillers of sizes below 50, 75, and 100 microns were used. The hemp fiber in polymer composites showed a significant influence in its water uptake behavior with the value of water absorptivity 2.41 × 10^−6^ g/m^2^.s^1/2^. The incorporation of biocarbon fillers in the hemp biopolymer composites reduces the average water absorptivity by 44.17% and diffusivity by 42.02%. At the optimized conditions, the value of water absorptivity with hemp biocarbon and switchgrass biocarbon fillers was found to be 0.72 × 10^−6^ g/m^2^.s^1/2^ and 0.73 × 10^−6^ g/m^2^.s^1/2^, respectively. The biocarbon at 650 °C showed the least composite thickness swelling due to its higher porosity and lower surface area. Biocarbon-filled hemp composites showed higher flexural strength and energy at the break due to the enhanced mechanical interlocking between the filler particles and the matrix materials. Smaller filler particle size lowered the composite’s water diffusivity, whereas the larger particle size of the biocarbon fillers in composites minimizes the water absorption. Additionally, higher filler loading results in weaker composite tensile energy at the break due to the filler agglomeration, reduced polymer-filler interactions, reduced polymer chain mobility, and inadequate dispersion of the filler.

## 1. Introduction

Only in Canada, over 3 million tonnes of plastic waste, accounting for more than 80%, end up in landfill [1]. This issue can be tackled by biobased materials to ensure a better, cleaner, and sustainable future [2,3,4]. Natural fiber-based biocomposites research, development, and promotion of plant-based materials help primarily complement the fossil fuel source produced and to reduce and replace the plastics in the long run [5,6]. Among the available plant fibers, hemp is known for its lightweight and better mechanical strength leading to an impressive strength-to-weight ratio [7,8]. Its quicker growth and resilience to extreme weather add value to production and promotion [9,10]. However, plant-based materials suffer from the disadvantages such as their hydrophilicity which enable them to readily absorb moisture compromising their mechanical and other physical properties and resulting in reduced longevity [11,12]. Water negatively influences material cost as it reduces the green material’s service life and overall performance. Hemp fiber has shown similar properties compromise due to the water absorption phenomena when used in a moisture-prone environment [13,14]. Therefore, the service life of reinforcing hemp fiber can be improved if its water absorption behavior is altered and reduced.

There have been studies on applications of filler materials in polymer composites to stabilize, improve, enhance, and optimize the materials’ water performance [15,16]. It has been found that the filler in composite materials improves their mechanical properties, thermal conductivity [17], and physical characteristics. For instance, rice husk, walnut shell, and coconut shell in the form of particulate fillers in bio-epoxy resin and hardener were studied by Chandramohan D. and John PKA [18] to investigate the mechanical properties and water’s effect on the mechanical strength of the composite samples. The hybrid composite samples showed better flexural strength in wet conditions than the dry samples. Walnut- and coconut-containing samples showed minimum water absorption and superior tensile strength (68.8 MPa), flexural strength (14.9 MPa), and shear strength (81.92 MPa). The value for the elongation at break (21.82%), energy absorbed during the impact test (20.9 MPa), and breaking load for tensile and flexural tests of the composite samples with walnut and coconut shells were also better than those of the composites with rice husk and coconut shell fillers and rice husk and walnut shell fillers. The water buffering capacity, mechanical properties, and thermal properties of hempcrete (hemp fiber in concrete) were studied by Abdellatef Y. et al. [19]. Similarly, biocarbon in polymer composite materials [20,21] may add cost-effectiveness and environment friendliness to the materials. Thus, biocarbon can be utilized to tailor-make newer, lighter, and sustainable composite materials [22,23]. Biocarbon is the product obtained by removing the volatiles from the biomass during its thermal degradation process. The hemicellulose, cellulose, and lignin present in the biomass samples are decomposed throughout this process [24]. The carbon concentration in the biocarbon improves by increasing the pyrolysis temperature in a controlled and inert environment. During the pyrolysis process, the removal of -OH bonds from the biomass results in a stable and hydrophobic biocarbon [25]. Additionally, releasing volatiles from the biomass leaves a void inside the biomass structure, creating hexagonal space [26,27]. This process causes higher porosity in the biochar structure. Microporous (below 2 nm), mesoporous (between 2 to 50 nm), and macroporous (above 50 nm) can be achieved in biocarbon by changing the process temperature, residence time, and heating rate [25]. The pore size is of greater importance as it helps maintain the flow of liquid and gas through it, the inclusion of matrix within it and the filtration of different-sized particles. The stable and hydrophilic biocarbon with a honeycomb structure for easy flow of resin influences the mechanical properties and water absorption nature of the resulting composite material when used as a natural filler material in polymer composite [28,29].

Gupta et al. (2017) found that the biocarbon in polypropylene results in a significant reduction in water penetration by at least 35% compared with the control sample [30]. On the contrary, biocarbon in concrete caused an increase in water absorption by a small amount of 0.8% compared to biocarbon-free concrete [31]. Prabhu et al. (2022) showed that the biocarbon inclusion (up to 7%) in epoxy composites significantly reduced their contact angle preserving their hydrophobicity due to the increase in surface energy in the composite’s surface [32]. In another experiment, Zhang et al. included 150 µm biocarbon filler obtained at 600 °C ranging from 10% to 70% in HDPE, concluding that 70% is the maximum filler loading in the polymer composite. They found that the water absorption is proportional to the biocarbon loading [33]. The literature search shows greater potential for biocarbon to be used as a polymer filler, but their influence due to the particle size and filler loading in the composite materials is limited.

The authors find a research gap to study the interaction effect of the design parameters, such as the filler size, filler loading, and the pyrolysis temperature on the physical properties of the composites. Besides, the application of biocarbon in the hemp-reinforced biopolymer composite is limited. This work presents a novel concept of developing a bio-based material in which the polymer, fiber, and filler are all sourced from plants. This paper adds valuable information in the biopolymer–filler–fiber composite field and offers the interaction effects of the design parameters on the hemp composite material’s properties.

In this work, the hemp-reinforced biopolymer composite is filled with biocarbon of various particle sizes and filler loading. The biocarbon is obtained at three different temperatures from hemp and switchgrass plants. The pyrolysis temperature in the range of 450 to 650 °C is chosen because this range of temperature is optimal for breaking down the complex organic molecules in biomass into simpler pyrolytic products. At a temperature lower than 450 °C, the pyrolysis reaction is slower and incomplete, which will result in a low yield of useful products. At temperatures higher than 650 °C, the biomass decomposes rapidly, resulting in the production of a high proportion of non-condensable gases, including CO and hydrogen. Controlled pyrolysis at a temperature range of 450 to 650 °C results in an optimized pyrolysis process with a high yield of desirable pyrolytic products: biocarbon, bio-oil, and syngas. Therefore, the temperature range of 450–650 °C was chosen for this experiment.

Additionally, a comparative study is presented to understand the behavior of different plant-sourced biocarbon in biopolymer composites. The influence of biopolymer composites’ water absorptivity on their mechanical properties is studied. Finally, the biocomposites were optimized for their formulation based on their water absorption behavior. An optimized biocarbon formulation (loading, particle size, and pyrolysis temperature) with a full-factorial design of experiments is performed.

## 2. Results and Discussion

### 2.1. Biocarbon Characterization

#### 2.1.1. Physiochemical Analyses of Biocarbon

The samples’ moisture, fixed carbon, ash, and volatile matters were determined by proximate analysis of the raw hemp and switchgrass samples and hemp and switchgrass biochar samples. The results from the physiochemical analyses have been presented in the Supplementary Datasheet. Results show 5 to 7% moisture content in the raw biomasses, and the value was substantially reduced in all the biochar samples after the pyrolysis. The volatile matter content (dry basis) in raw hemp and switchgrass biomass was 73.34% and 82.71%, respectively. Similar results were obtained during the proximate analysis of the switchgrass [34] and hemp stalk [35] and pine, rice husk, and wheat straw [36]. The trend of fixed carbon and the volatile matter was reciprocal against the temperature.

As expected, the fixed carbon increased from below 10% in raw samples to above 60% in biochar samples. The high percentage of volatile matter in raw samples ranging above 70% in hemp and above 80% in switchgrass was removed significantly in biochar samples obtained at higher temperatures. This increase in carbon content was attributed to the subsequent reduction of nitrogen, hydrogen, and oxygen in the form of volatile matter. During the pyrolysis process, the weaker bonds containing mainly C and O were broken to form a more robust carbon structure in biochar [24]. This resulted in higher carbon content in the biochar samples compared to their respective raw biomass samples. The ash content in biomass samples was below 5% (dry basis). The higher pyrolysis temperature reduced the greater amount of volatile matter, leaving a higher proportion of ash in the biochar samples.

During the analysis, fixed carbon was determined from the difference. As expected, there was a rise in fixed carbon proportion as the pyrolysis temperature was increased in both feedstocks due to the removal of moisture and volatile matter.

**Figure 1:**
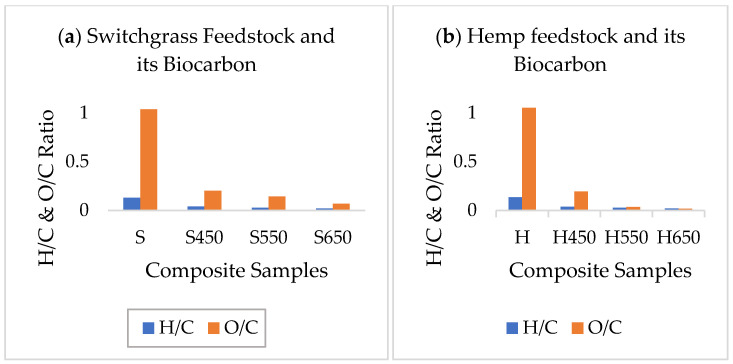
H/C and O/C trend in (**a**) switchgrass feedstock and its biochar, and (**b**) hemp feedstock and its biochar at different pyrolysis temperatures.

The H/C ratio estimates the aromaticity, and the O/C ratio represents the polarity of the biochar; the lower the value of H/C, the higher the aromaticity and the degree of carbonization [37,38]. The ratios in the biochar samples were lower than their parent biomass feedstock. The lower H/C ratio also suggests that there is less amount of original organic and unsaturated residuals remaining, which were dehydrated and thermally altered due to high pyrolysis temperature [39]. The ratios further declined with the increase in pyrolysis temperature, as shown in Figure 1a,b. This was due to the removal of volatile matter, mainly containing hydrogen and oxygen. Results from the analyses are provided in Supplementary Information (Table S1).

#### 2.1.2. FTIR Analysis

**Figure 2:**
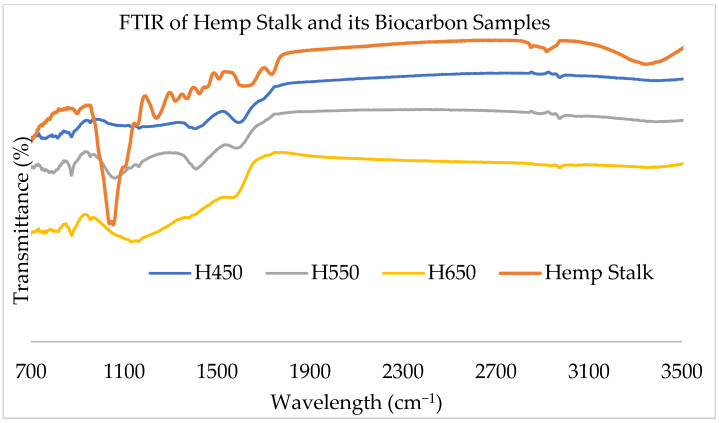
FTIR of the hemp stalk and its biocarbon at different pyrolysis temperatures.

**Figure 3:**
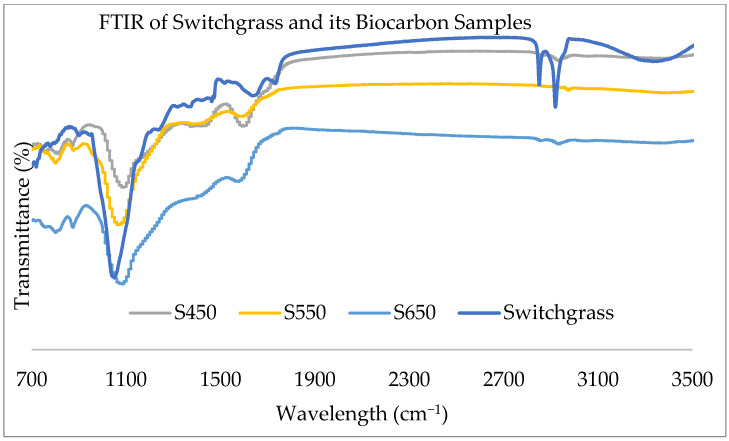
FTIR of the switchgrass and its biocarbon at different pyrolysis temperatures.

The aim of the FTIR is to characterize the biomass samples and their respective biocarbon samples to see the change in chemical composition, functionalization, and transformations in the samples. The comparative infrared spectroscopy of the hemp and switchgrass biomass and biocarbon samples at different pyrolysis temperatures are given in Figure 2 and Figure 3. The comparative IR spectra show that the noise in raw biomasses at 3360 cm^−1^ has been eliminated in the biocarbon samples. This is due to the removal of the O-H stretching in hemicellulose, cellulose, and lignin. This also indicates the elimination of unstable alcoholic, phenolic, and hydroxyl groups at higher temperatures during the dehydrogenation process in pyrolysis. Another noticeable removal of the peaks at higher temperatures, especially at 650 °C, was observed at 2920 cm^−1^ due to the removal of the C-H stretching in hemicellulose, cellulose, and lignin mainly due to the reduction of waxes and aliphatic CH stretching vibration due to the removal of the weaker CH bond of the alkyl groups. At 1730 cm^−1^, absorption in switchgrass was due to the stretching of C=O in ketone/aldehyde in hemicellulose [40], pectin, and waxes [41]; additionally, absorption in hemp stalk at 1750 cm^−1^ is due to pectic acid and free ester in hemicellulose [40]. At 1650 cm^−1^, the absorption was the result of the unconjugated C=O stretching [42] and the OH bending of absorbed water [41] in both biomass samples. In hemp, the aromatic ring vibration and C=O stretching in lignin [40] present caused absorption at 1600 cm^−1^. Similarly, absorption at 1520 cm^−1^ was due to the C=C aromatic ring vibration present in lignin [40,41] in both raw samples. The C-H deformation in lignin [40] was absorbed in 1465 cm^−1^ in both raw samples. The C-H in-plane deformation and O-H in-plane bending in cellulose and lignin absorbed the radiation in the hemp stalk at 1430 cm^−1^ [40,41]. Both feedstocks showed absorption at 1380 cm^−1^ due to the C-H bending in cellulose, hemicellulose, and lignin [40]. The presence of the C-H bond of the syringyl ring in lignin [42] and CH2 rocking vibration in cellulose [41] caused noise at 1320 cm^−1^ in hemp stalk. The absorption at 1300–600 cm was due to the presence of low molecular weight carbohydrates, polyols, and monosaccharides [43,44]. The raw samples contained C-C and C-O stretching [42], aromatic ring vibration in Guaiacyl lignin [40], and C=O and G ring stretching in lignin [41] at 1240 cm^−1^. At 1200 cm^−1^, switchgrass showed absorption due to the symmetrical stretching of C-O-C [41] and bending of O-H in cellulose and hemicellulose [40]. Both samples absorbed radiation at 1160 cm^−1^ due to the asymmetrical stretching of C-O-C in cellulose and hemicellulose [40,41]. Absorption in hemp stalk at 1110 cm^−1^ was due to the symmetrical stretching of C-O-C in ester groups present in cellulose and hemicellulose [45,46] and in-plane deformation of aromatic C-H in lignin [47]. The IR spectra showed absorptions in both samples at 1050 cm^−1^ due to the C-O, C=O, and C-C-O stretching in cellulose, hemicellulose, and lignin [40,41] and symmetrical stretching of C-O-C in aliphatic groups and acid derivatives [45]. Glycosidic linkage in cellulose and hemicellulose resulted in the absorption in IR spectra in both samples at 930 cm^−1^ and 860 cm^−1^ [40]. C-O-C, C-C-O, and C-C-H deformation and stretching in cellulose peaked at 900 cm^−1^ in switchgrass [41]. Absorptions at 850 cm^−1^ and 833 cm^−1^ were associated with out-of-plane bending of aromatic C-H in phenolic compounds [47] and out-of-plane bending of lignin [46], respectively. CH2 rocking bending in waxes present in samples is attributed to the peaks at 720 cm^−1^ [46]. Both samples experienced absorption at 660 cm^−1^ due to the C-OH out-of-plane bending in cellulose [41]. 

The resistant aromatic carbon groups and aliphatic CH2 groups in lignin even at increased pyrolysis temperatures caused peaks in 1400 cm^−1^ [45]. The presence of C=O stretching of methyl ester and carboxylic acid in pectin (containing both esterified and carboxylic acid groups) [48] and waxes, hemicellulose, cellulose, and lignin responsible for the absorption at 2850 cm^−1^ [49] in raw samples were removed in the biocarbon samples. Similarly, the preserved aromatic ring, the C=O stretching in lignin [40], and the presence of polyphenols [50] in the biocarbon samples caused the absorptions at 1600 cm^−1^. The biocarbon samples also showed the presence of aromatic C-H in lignin [47] associated with the absorption at 1110 cm^−1^. The reappearing absorptions in biocarbon at 850 cm^−1^ were due to aromatic C-H in phenolic compounds [47]. The shifting of the baseline towards the left in the biocarbon samples at higher temperatures is due to the loss of functional groups and improved graphitization of the biocarbon samples [51].

#### 2.1.3. SEM Analysis

**Figure 4:**
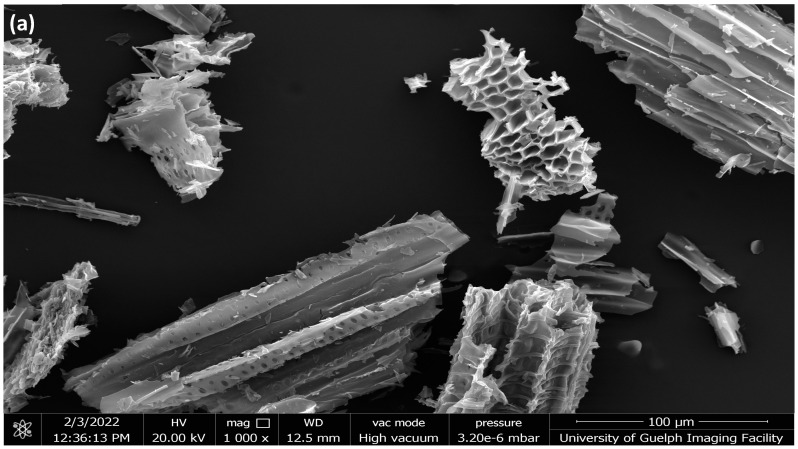

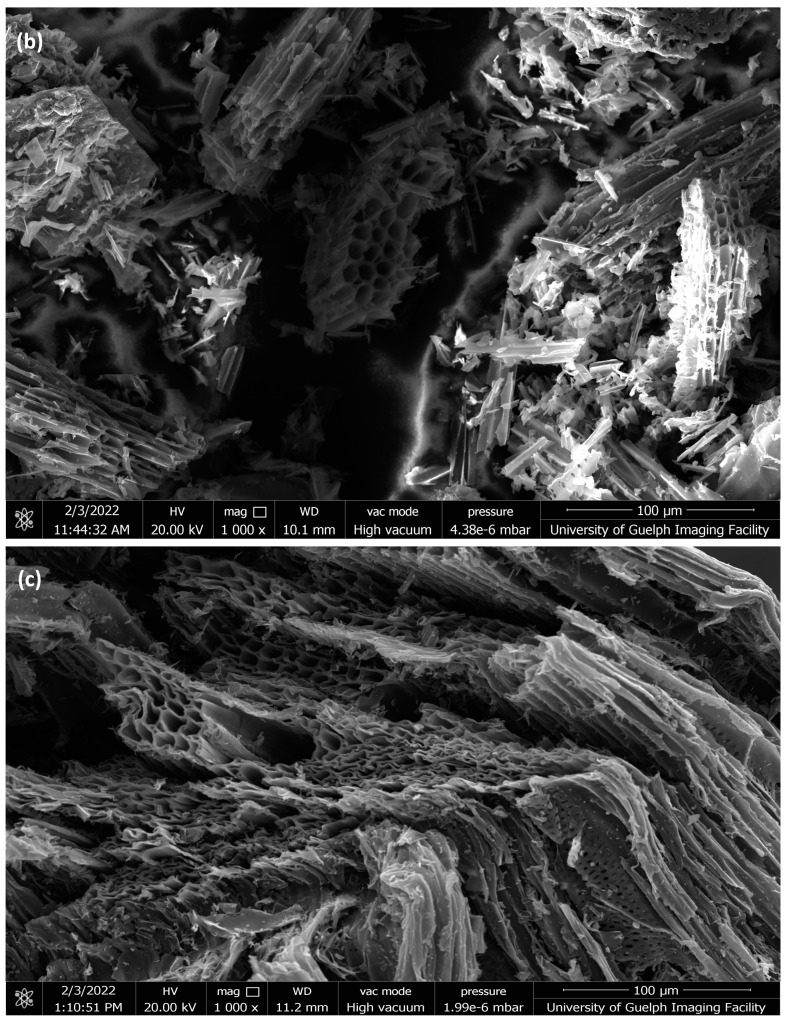
SEM images (200 µm) of hemp biocarbon obtained from pyrolysis at (**a**) 450 °C, (**b**) 550 °C, and (**c**) 650 °C, respectively.

**Figure 5:**
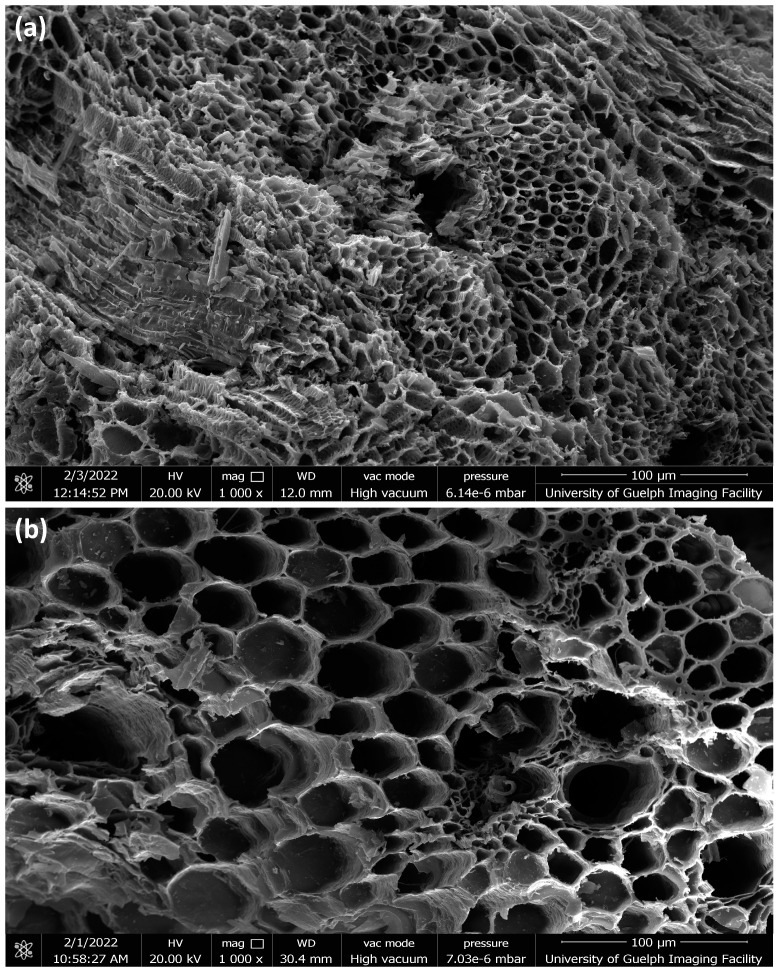

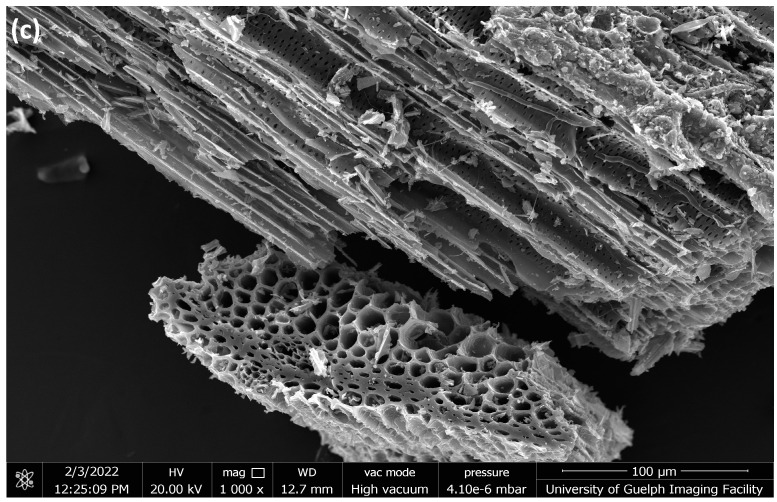
SEM images (200 µm) of switchgrass biocarbon obtained from pyrolysis at (**a**) 450 °C, (**b**) 550 °C, and (**c**) 650 °C, respectively.

Figure 4 and Figure 5 show the SEM images of the hemp and switchgrass biocarbon obtained at 3 different pyrolysis temperatures: 450 °C, 550 °C, and 650 °C. The images show the porous structure present in all biocarbon samples. These hexagonal voids are left in the remaining biocarbon due to the release of the volatile matters present in the original biomass. The escaping of such volatile matters creates pores and cracks on the biocarbon surfaces [52].

### 2.2. Explanations on the Composites’ Water Absorptivity and Diffusivity

**Figure 6:**
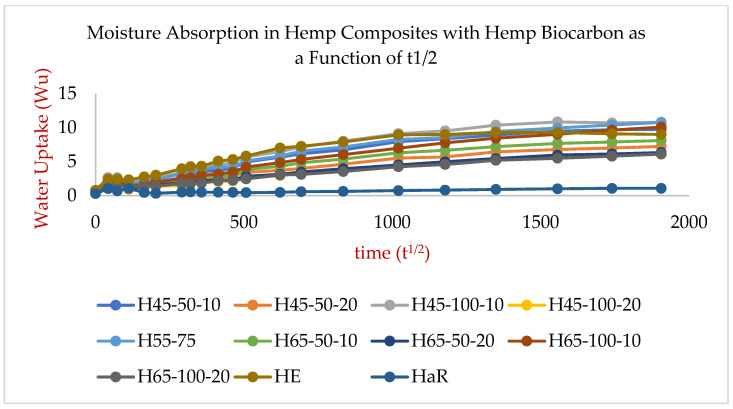
Water uptake nature of various hemp-biochar composites as a function of the square of time.

**Figure 7:**
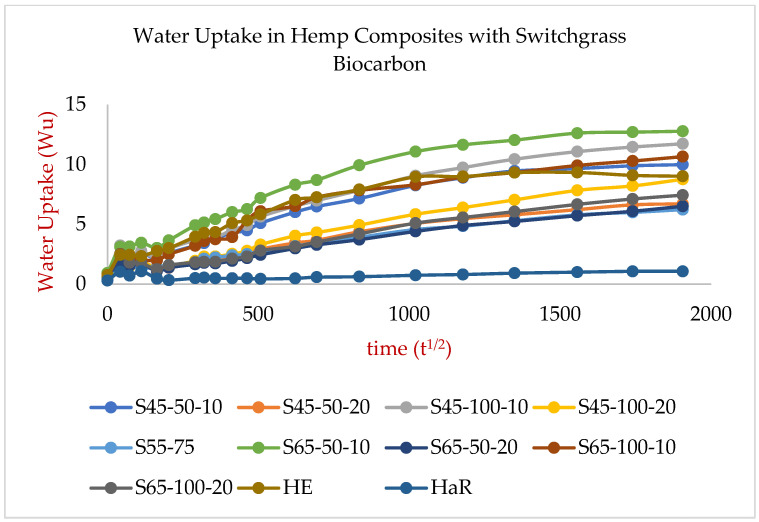
Water uptake nature of various switchgrass-biochar composites as a function of the square of time.

The results from the water absorption test that offered the water absorption behavior of the hemp-reinforced hemp biocarbon-filled biopolymer composite samples have been presented in Figure 6, and those of the hemp-reinforced switchgrass biocarbon-filled biopolymer composites are presented in Figure 7.

Among the theoretical and empirical models to study the water uptake nature of a material, empirical law based on Fick’s law is preferred as the theoretical models include functions and parameters impractical for calculations. Among the models obeying Fick’s law, the water absorption coefficients of the samples were calculated applying the following equation as per the ISO 12572:2016 [53,54] that is derived by implementing the Bernoulli equation of hydrodynamics and the Hagen–Poiseuille law for flow through tubes:

W_u_ = A_c_·√t

Hence,

A_c_ = W_u_/√t

where W_u_ is the water uptake in g/mm^2^, and A_c_ is the water absorption coefficient (s^1/2^. g/mm^2^). A_c_ is obtained from the slope of the fitted curves and dividing the slopes by the samples’ surface area (S). The curves from the percentage of the mass of water uptake per unit surface area as a function of the square root of time for hemp are linear for the first week (first 60 h) of measurement, as shown in Figure 6 and Figure 7. The values of the average water absorption coefficients (A_c_) of the hemp composites along with their water diffusivity values (D_t_) have been presented in the Supplementary Datasheet. The capillary water absorption of the samples with lower biochar concentrations was seen to be significantly higher as compared to the samples with higher biochar concentrations Figure 6 and Figure 7 show that the composite with higher biochar concentrations shows slower water uptake. The initial higher water absorption rate is due to the water diffusion phenomenon due to the fluid migration and its spread through capillaries, vessels, and cell walls in the samples. The rate of water migration at any time is directly proportional to the difference in water content at saturation and water content at that specific time. 

The diffusion of water in solid samples of any arbitrary shape during their soaking in water is given by [55]:

(M_t_ − M_o_)/(M_s_ − M_o_) = 2/√π·(S/V)·√(D_t_·t) = A_c_·√t

Hence,

D_e_ = π/4·(V/S)^2^·(A_c_)^2^

where M_t_ is the water uptake at time t, M_o_ is the initial water uptake, M_s_ is the water uptake at saturation point, D_e_ is the water diffusivity of the composite materials, V is the volume, and S is the surface area of the composite samples.

The water absorption trend in all samples shows a rapid rise in water absorption initially that is followed by an intermediate water absorption rate until the relaxation phase at saturation point is reached [56,57], as shown in Figure 6 and Figure 7. The water uptake in the first 4 days (~108 h) was almost about half the total water absorbed by the samples. After that, the water absorption was slow until the samples were fully saturated. The capillaries and cavities on the surface are filled readily, resulting in the rapid rise of the water uptake for the first couple of days. The water travels slowly inside the material due to the restricted small passages and the apparent vacuum created by the water surface on all sides of the voids. J. Khazaei (2007) [56] found that the maximum water uptake (~60%) in Afra, Ojamlesh, and Roosi woods took place in a short period (2 days); the author found the water diffusion of coefficients for the woods were 1.38 × 10^−3^, 3.71 × 10^−4^, and 4.88 × 10^−4^ m^2^/s, respectively. The water absorption and diffusion in particle-filled and hemp-reinforced samples took longer to saturate than the HaR sample due to the existing longer routes created by the particles and fibers [58]. 

**Figure 8:**
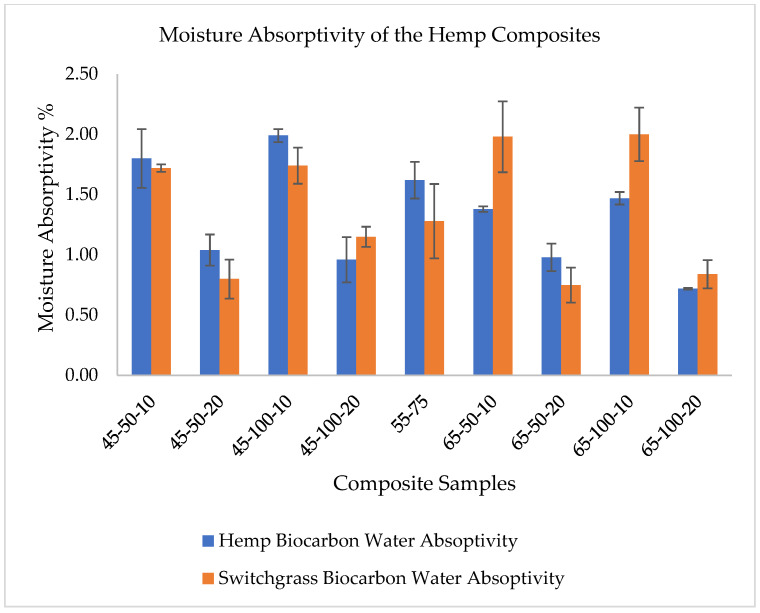
Difference in mean water absorptivity of various composite samples.

Table S2 in Supplementary Data summarizes the ANOVA test of the water absorptivity and water diffusivity of the composite samples with hemp and switchgrass biocarbon fillers and Table S3 provides the values for the bio-epoxy samples without biofiller and fiber. Boxplot Figure 8 shows the significant differences in mean. The ANOVA result on the water absorptivity of hemp biocarbon composites showed F statistics (F-value = 12.18) with a very small *p*-value (*p* = 6.85 × 10^−8^), suggesting solid evidence against the null hypothesis that all true means of water absorptivity of composites containing hemp biochar are equal at a chosen significance level of 0.05. The F-statistics (F = 12.19) with a very small *p*-value (6.64 × 10^−5^) for the analysis of water diffusivity of the composite samples with hemp biocarbon provides strong evidence in support of the alternative hypothesis that at least 1 of the true mean pairs of the different hemp biocarbon containing composite samples does not have the same true mean water diffusivity at a significance level of 0.05.

A similar result was obtained from the ANOVA test of the water absorptivity and water diffusivity of the composite samples with switchgrass biocarbon filler. The F-statistics (F-value = 19.27) with a very small *p*-value (*p*-value = 4.85 × 10^−8^) for the means of water absorptivity and F-statistics (F-value = 21.58) with a very small *p*-value (*p*-value = 1.75 × 10^−8^) indicates that there is at least a pair of varying true means in water absorptivity and water diffusivity of the switchgrass biocarbon-filled hemp fiber-reinforced biopolymer composites.

The water diffusivity of the composite samples without fiber and biofillers is almost negligible, while the water diffusivity of the hemp-reinforced composite is the greatest among all composite samples [58]. The main reason for the superior water diffusivity is the water-sucking ability and the ability to quickly transmit moisture through the capillary action utilizing the cell walls and cavities of the natural hydrophilic fibers. This capillary action is the main reason for the HE composites’ significantly higher water absorption behavior than the HaR and other composites. The quicker water uptake also resulted in a faster saturation of the composite sample, as seen in Figure 6 and Figure 7. The diffusivity and the water absorptivity nature of the resin–hardener composite were close to zero. The homogeneity of the resin–hardener composite material is responsible for its low water absorptivity, which caused a reduction in its porosity [59]. In the switchgrass biocarbon-filled composites, the composites’ water absorptivity increased by 8.7% when the particle size was increased by 50% from 50 µm to 75 µm as shown in Figure 8. However, a 100% increase in particle size from 50 µm to 100 µm did not affect the water absorption significantly, causing only 2% of the water absorption. This low water absorption at higher particle sizes can be attributed to the reduction of gaps with better adhesion between the matrix and the larger-sized filler particles [60]. The 100 µm sized filler particles in the composite create a more compact and dense structure, which reduces the amount of void space available for water to penetrate the composite material. This reduces the available space for water to penetrate the composite and hence reduces the amount of water that can be absorbed. Additionally, the 100 µm filler biocarbon particles can create a barrier that prevents water from penetrating deeply into the matrix, further reducing the amount of water absorption. It is important to note that the choice of filler particle size should be carefully balanced with other factors, such as the desired mechanical properties, processability, and cost of the composite material.

### 2.3. Water Absorption Effect on the Thickness Swelling of the Composites 

**Figure 9:**
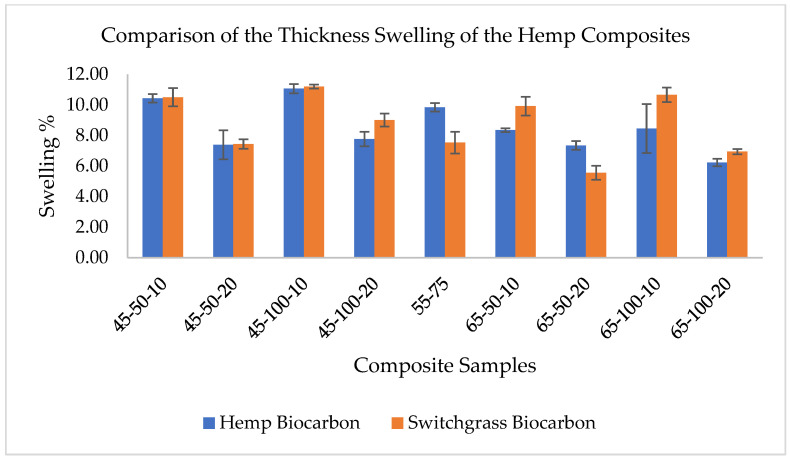
Thickness swelling of hemp reinforced composite samples with and without biocarbon filler.

The composite samples were analyzed for their swelling behavior due to the water absorption effect. The increase in thickness due to the water uptake was an indicator of the swelling effect in the composite samples. The findings from the experiments are presented in Supplementary Datasheet. A similarity in the thickness due to the water absorption of the composite samples was observed. Figure 9 shows the boxplot of the swelling percentage of the biocomposite samples. The average swelling percentages of the biocomposite samples are provided in Supplementary Table S4.

There is almost negligible effect on swelling of the composite with no fiber and no filler loading (HaR), and there is a maximum swelling in the composite (HE) with hemp fiber but no biocarbon in it. The thickness swelling behavior of composite samples containing fibers and fillers is due to the hydrophilic nature of the natural fibers that readily absorb the water. The water uptake by the fiber’s cell walls and the hydrophilic nature of the cellulose in the fibers result in the swelling nature of the composite as compared to the pure epoxy-resin composite (HaR). Firstly, the fiber added to the polymer results in ramping thickness swelling due to the hydrophilic behavior of the plant-based fibers. Like in water absorption curves, added biocarbon in the hemp polymer composite reduces the swelling. The swelling was further suppressed by increasing the biocarbon filler in the composites. The trend was similar in both composite samples with hemp biocarbon and switchgrass biocarbon. Biocarbon fillers at higher temperatures caused lesser thickness swelling due to water because of their higher porosity and lower surface area, which reduces their ability to absorb water and swell. The more stable carbon-rich biocarbon with a more ordered structure with fewer functional groups on the surface due to the pyrolysis at higher temperatures reduces the number of sites available for water molecules to bind to, which reduces the fillers’ ability to absorb water. Biocarbon produced at higher temperatures is less absorbent and, therefore, less prone to swelling, which improves the dimensional stability and mechanical properties of the composite materials.

From the ANOVA test, there is very strong evidence (*p*-value = 7.7 × 10^−4^) that not all composite samples with hemp biocarbon have the same population mean swelling percentage. A similar low *p*-value = 4.85 × 10^−8^ infers that at least a pair of composites with switchgrass biocarbon have different mean swelling percentages.

### 2.4. Water Absorption Effect on the Mechanical Properties of the Composite Materials

**Figure 10:**
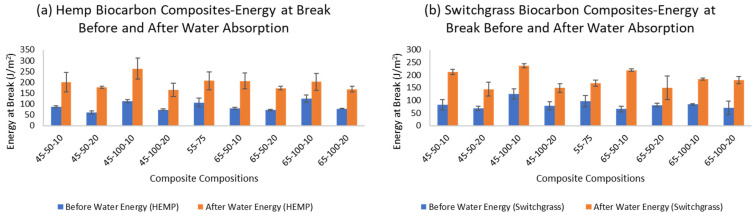
Energy at break of hemp composites before and after the water absorption test with (**a**) hemp biocarbon and (**b**) switchgrass biocarbon.

The energy at the break of a composite material (fracture energy) represents the work done per unit area to fracture the material. The energy at the break of the hemp-reinforced composite samples is presented in Figure 10 and the data are provided in Table S6.

During the tensile test, the unfilled unreinforced resin hardener (HaR) composite demonstrated the energy at break value of 119.54 J/m^2^. Similarly, the value of the hemp-reinforced unfilled polymer composites was 137.57 J/m^2^, the highest among all the tested composite samples. These expected results of improved mechanical properties with natural fiber addition have been presented by Awad et al. (2022) [61]. The hemp-reinforced polymer composite adds fracture resistance to the samples. The energy at break almost doubled in the composite samples except for the unfilled unreinforced composite, for which the value remained unchanged even after the water absorption. The increasing energy at break due to water absorption in the composite materials was mainly due to the moisture acting as a plasticizer. The water molecule in between the polymer chains weakens the intermolecular forces that make the samples more flexible and easier to deform, which in turn increases the material’s strain capacity allowing it to absorb more energy before failure. Another reason can be due to the water causing microcracking due to its penetration into the composites causing fiber swelling and resulting internal stresses in the material. These microcracks helps dissipate energy and increases the composite material’s toughness and energy absorption capacity. Hydrolysis may occur when moisture reacts with the polymer matrix weakening the polymer, reducing the stiffness, and increasing the strain capacity and, thus, energy absorption capacity.

The one-sided paired *t*-test in hemp biocarbon composites indicated strong evidence (*p*-value 4.26 × 10^−12^) that the true mean difference in the energy at break is greater than 0. Since there is strong evidence that the true mean difference in the energy at break is greater than zero, it implies that the water absorption increases the energy at break of the composite samples. The result from the tensile test shows that the biocarbon addition in the hemp-reinforced polymer composite samples reduces the energy at the break. The higher filler loading in the composite samples reduced the energy required to rupture, while the particle size also influenced the energy at the break in a different way. The 100 microns particle-sized biocarbon in the composite samples increased the breaking energy in all the samples. The three-point bending test was performed to study the flexural properties of the hemp-reinforced composite samples. The data from the flexural test showing the average flexural modulus of the samples are presented in the Supplementary Information Sheet.

**Figure 11:**
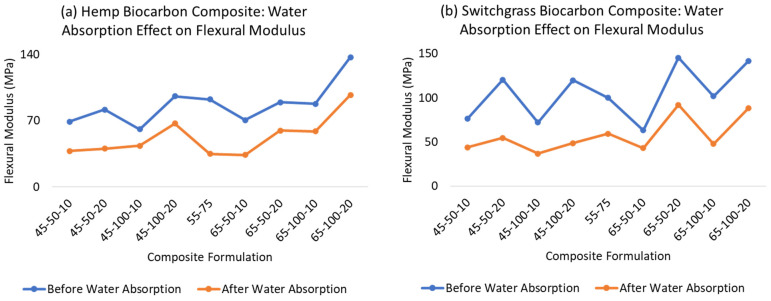
Difference in mean flexural moduli of biocarbon-filled hemp composite due to water absorption.

Similarly, one-sided paired *t*-test in switchgrass biocarbon composites provided strong evidence (*p*-value = 3.86 × 10^−15^) that the true mean difference in flexural modulus (i.e., flexural modulus before water absorption minus flexural modulus after water absorption) is higher than 0. The point estimate of the true mean difference in the flexural modulus is 43.84 MPa, with a corresponding 95% confidence interval of 37.76 MPa and above. Therefore, it can be inferred with strong evidence that the water absorption decreases the flexural modulus of the composite samples. The difference in flexural properties is well illustrated in Figure 11 from the data provided in Supplementary Table S5.

When tested dry, the composite without fiber (HaR) showed the least flexural modulus of 36.60 MPa. However, its value remained almost unchanged after being fully saturated in water. The hemp biocarbon loading positively affected the flexural modulus of the hemp-reinforced composite samples. A similar inference can be deduced from the switchgrass biocarbon containing hemp composites. In contrast, the temperature change had the most negligible effect on the flexural properties of the composite samples. The wet samples performed very poorly in terms of flexural strength. It is observed that the water in the composite samples diminished the flexural strength of the material [58]. The flexural behavioral trend of the wet samples was similar to those of the flexural properties of the dry samples.

### 2.5. Biocarbon-Filled Composites’ Water Absorptivity Design of Experiments:

Table 1 presents the water absorptivity of the hemp-reinforced composite samples with biocarbon fillers. The water absorption property of natural fiber-based material is critical to its mechanical properties; the optimization analysis was performed based on the water absorptivity. Afterwards, the optimized composite samples are further studied for their mechanical and thermal behavior. Supplementary Figures S16 and S17 show the water absorption curves of hemp composites with hemp biocarbon and switchgrass biocarbon fillers respectively as a function of time (h).

**Figure 12:**
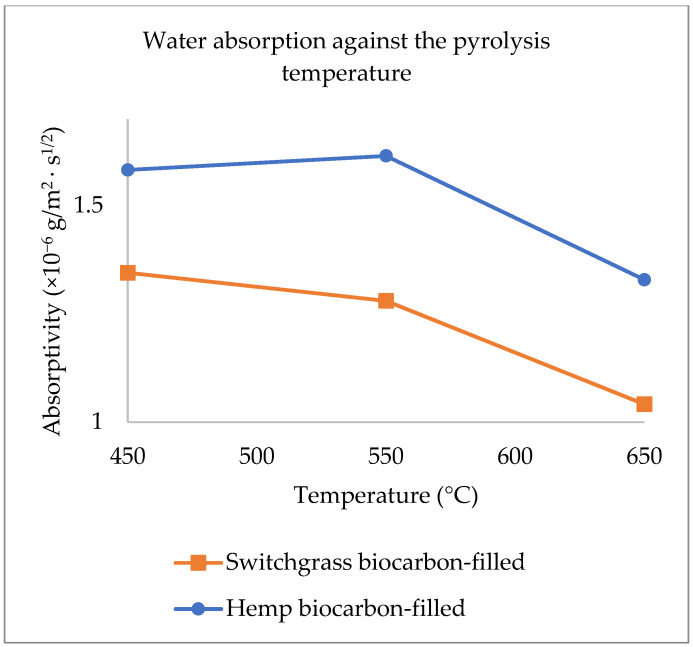
Main effect of the pyrolysis temperature on the water absorptivity.

**Figure 13:**
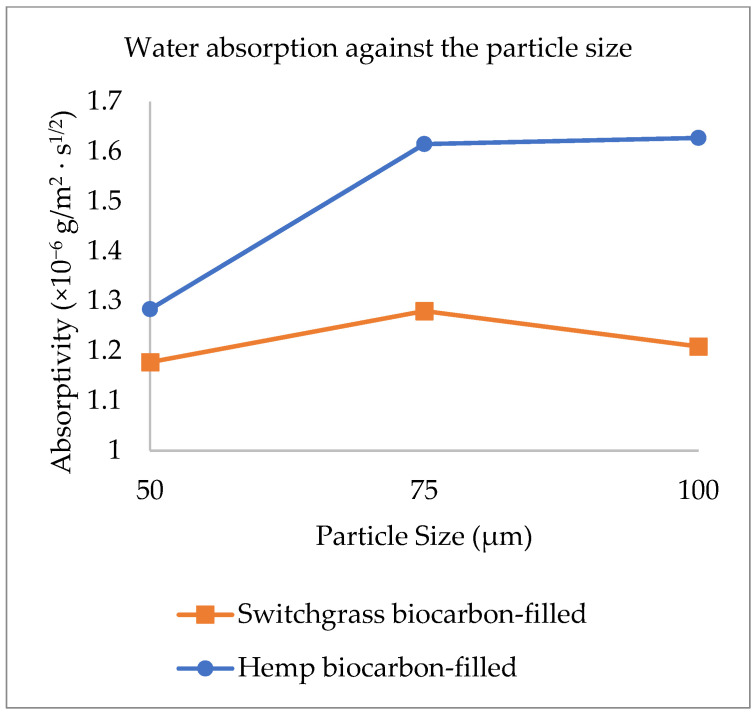
Main effect of the filler particle size on the water absorptivity.

**Figure 14:**
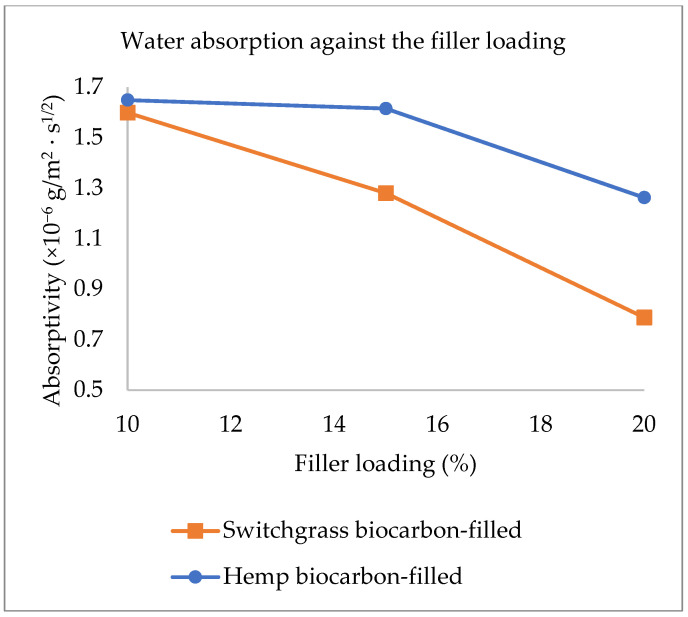
Main effect of the filler loading on the water absorptivity.

Mean water absorptivity is the response in the following Figure 12, Figure 13 and Figure 14.

#### 2.5.1. Biocarbon-Filled Composites Design of Experiments Based on Water Absorptivity

Water absorptivity as the response was analyzed on the Design Expert 12. The composite composition was determined to achieve the minimum water absorptivity of the hemp-reinforced biopolymer composite material. The hand layup technique and the limited pot time of the resin–hardener limited the further addition of the filler beyond 20% in the matrix. The biocarbon grindability posed a limitation to adding particles below 50 microns, and the experience from past research led us to choose filler loading between 10 to 20% and the filler particle size between 50 to 100 microns. The ANOVA is used to evaluate the multi-factor effect on the measured response (absorptivity of the composite samples). A three-way ANOVA with three factors was implemented for this study. The factors were changed at two levels, and a quadratic regression model was developed, including the interaction terms between and among factors. The effect of two different kinds of biocarbon (from hemp and switchgrass) on water absorptivity is discussed. 

#### 2.5.2. Statistical Analysis for the Biocarbon-Filled Hemp-Reinforced Biopolymer Composites

Design Expert 12 statistical software was used to analyze the significance of the effects of the factors with ANOVA. Table 2 shows a full model obtained from ANOVA considering all factors affecting the response.

The main effects of the variable factors on absorptivity are plotted in Figure 12, Figure 13 and Figure 14. In Table 3, considering the significance level (*p*-value = 0.05), the evaluation of the design of experiments on the Design Expert eliminated the B^2^ and C^2^ aliases terms for the hemp biocarbon-filled hemp-reinforced polymer composites. From Table 3, it is observed that when all sources are considered, the interaction effects do not significantly affect the water absorptivity of the hemp composites. There is no interaction between temperature and particle size (*p*-value = 0.6255) and no interaction effect of pyrolysis temperature and filler loading (*p*-value = 0.152) on the measured response for given levels of each factor. Additionally, the ANOVA analysis showed no 3-way interaction between the temperature, particle size, and biocarbon filler loading (*p*-value = 0.8763) at the given level of the factors. The curvature appeared insignificant (*p*-value = 0.3222), indicating that the design contains center points with a factorial model. Regardless, the Model F-value of 5.31 (*p*-value = 0.012) implies the model with hemp biocarbon fillers is significant, suggesting that there is only a 1.20% chance that an F-value this large could occur due to noise. However, for this full model, the predicted R^2^ of 0.2207 was not as close to the adjusted R^2^ of 0.6537 (indicating the difference is more than 0.2); possible reasons being a significant block effect or a possible problem with the model. Model reduction and response transformation and noticing the outliers are some of the possible solutions.

Similarly, for the switchgrass biocarbon-containing composite samples, the Model F-value of 14.35 (*p*-value = 00003) implies the model is highly significant, with only a 0.03% chance for an F-value this large to occur due to noise. In this model, the *p*-values less than 0.0500 indicate that the model terms are significant. Therefore, A (pyrolysis temperature) with *p*-value = 0.0117, C (filler loading) with *p*-value ≤ 0.0001, AB (interaction between the pyrolysis temperature and particle size) with *p*-value = 0.0117, and BC (interaction between particle size and filler loading) with *p*-value = 0.0211 are significant model terms. Here too, the curvature appeared insignificant (*p*-value = 0.5629), indicating that the design contains center points with a factorial model. For the full model, the predicted R^2^ of 0.6710 was in reasonable agreement with the adjusted R^2^ of 0.8538 (indicating the difference is not more than 0.2. The Fit Statistics for both (hemp and switchgrass biocarbons) composite types before the model reduction and elimination of non-significant terms are shown in Table 3.

After removing the curvature and eliminating the non-significant term for the hemp biocarbon-filled composites, i.e., A * B = interaction between temperature and particle size, A * C = interaction between temperature and particle size, B * C = interaction between particle size and particle loading, and A * B * C = three-way interaction between the pyrolysis temperature, particle size, and biocarbon filler loading, the ANOVA table for the reduced model for the water absorptivity as the response is presented in Table 4. Table 4 also contains the ANOVA table for the reduced model of the switchgrass biocarbon composites after removing the non-significant curvature and eliminating the insignificant terms, i.e., B (particle size), A * C = interaction between temperature and particle size, and A * B * C = three-way interaction between the pyrolysis temperature, particle size, and the filler loading.

From Table 4, it is inferred that the Model F-value of 10 with *p*-value = 0.0009 for the hemp biocarbon composites and F-value of 28.14 with *p*-value ≤ 0.0001 for the switchgrass biocarbon composites imply that the models are highly significant. There is only a 0.09% chance that the F-values these large could occur due to noise. The insignificant lack of fit for both models implies the lack of fit is insignificant relative to the pure error meaning that the model fits the data.

The regression equation from the reduced model for the water absorptivity of hemp biocarbon composites as the response was obtained as follows:

(Water Absorptivity) = 1.47 − 0.1269 * A + 0.1719 * B − 0.1931 * C

Similarly, the regression equation for the switchgrass biocarbon composites derived from the reduced model is as follows:

(Water Absorptivity) = 1.20 − 0.1519 * A − 0.4056 * C − 0.1519 * AB + 0.1344 * BC

The regression models with coded factors can be used to predict the response for given levels of each factor. The coefficient estimate represents the expected change in response per unit change in factor value when all remaining factors are held constant. The coded equation helps identify the relative impact of the factors on water absorptivity of the composite samples by comparing the factor coefficients.

#### 2.5.3. Model Adequacy Check

The figures for the model adequacy check for the biocarbon-filled composites are made available in the Supplementary Data. The data for the composite samples are normally distributed against residuals. It is seen that the residual plots follow a straight line to verify the normality assumption. Therefore, the plot meets the normality assumptions of the data. Similarly, the lack of fit test results in Table 5 shows that the model, along with the main and interaction effects, fits in the experimental data. The sum of squares of residual error was separated from the sum of squares of the pure error to show that the lack-of-fit is insignificant relative to the pure error, which suggests that the model is suitable to fit our data. In addition to the lack of fit test, the goodness of the model is further supported by the R^2^ statistics. The R^2^ value and adjusted R^2^ values show that the models reasonably fit the experimental data. The PRESS (prediction error sum of squares) was found to be 1.03 for the reduced hemp biocarbon composites’ model and 0.68 for the switchgrass biocarbon composites’ model. The predicted R^2^ is well within the accepted value, suggesting that the model can predict the water absorptivity of the composite samples for new observations. The R^2^ statistics for the regression model are presented in Table 5, and the residual plots are presented in the Supplementary Data.

**Figure 15:**
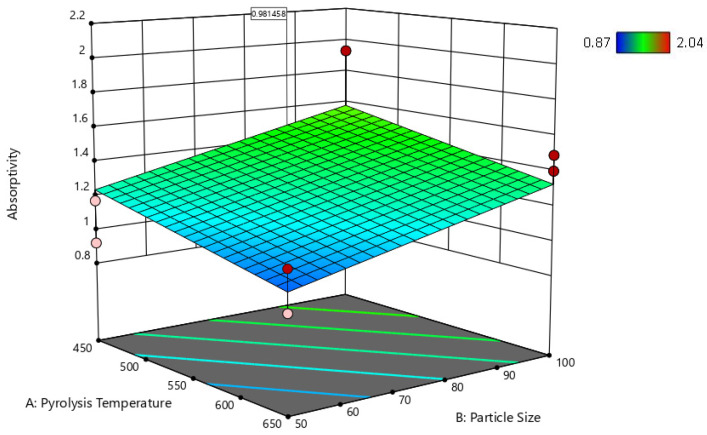
Surface response of the water absorptivity of the hemp biocarbon composites as a function of pyrolysis temperature and the particle size when the particle loading is 20%.

**Figure 16:**
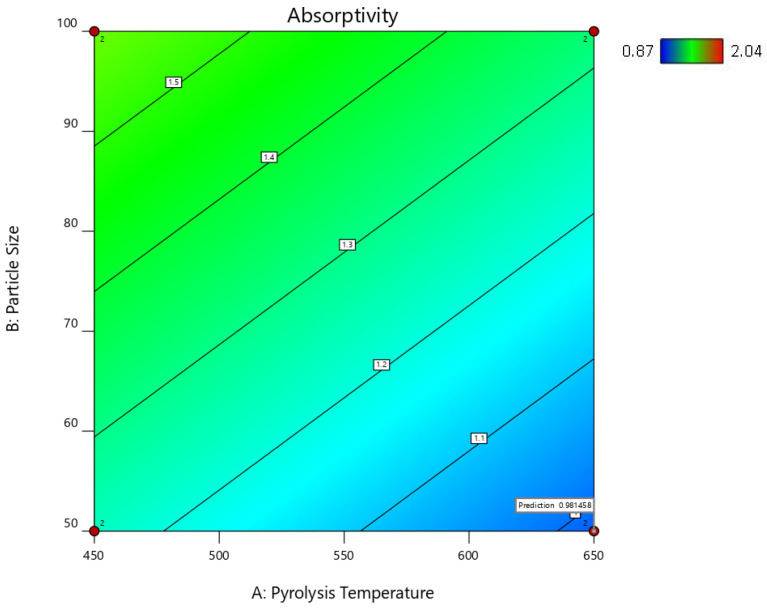
Contour plot of the water absorptivity of the hemp biocarbon composites against the particle size and the pyrolysis temperature when the filler loading is 20%.

**Figure 17:**
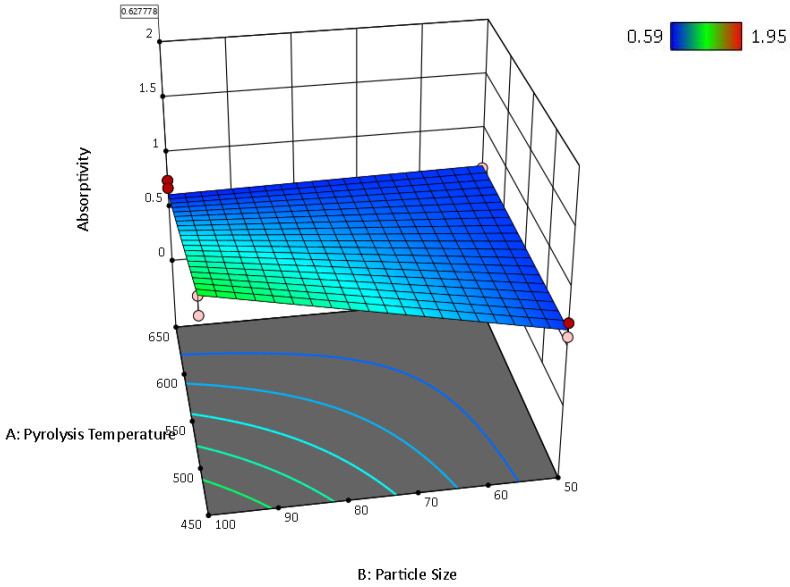
Surface response of the water absorptivity of switchgrass biocarbon-filled composites as a function of pyrolysis temperature and particle size when the particle loading is 20%.

**Figure 18:**
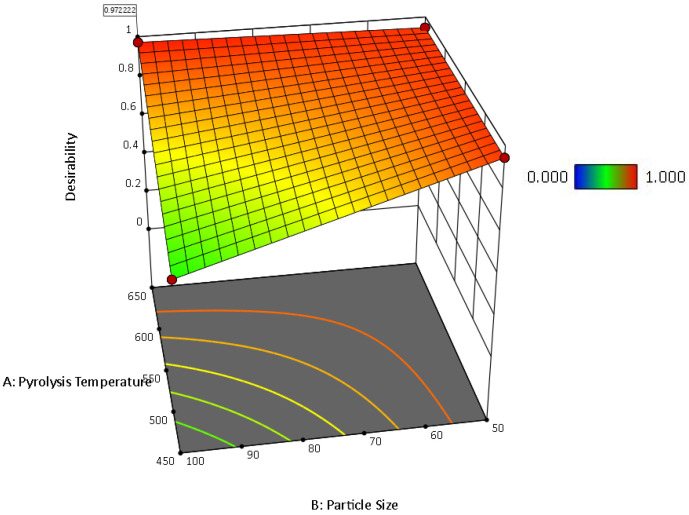
Surface response of the desirability of the water absorptivity of switchgrass biocarbon-filled composites as a function of pyrolysis temperature and particle size when the particle loading is 20%.

Table 5 shows the predicted R^2^ of 0.4728 for the hemp composites, which is in reasonable agreement with the adjusted R^2^ of 0.6136; the difference is less than 0.2. Additionally, for the switchgrass biocarbon composites, the difference between the predicted R^2^ (0.8329) and the adjusted R^2^ (0.8646) is less than 0.2, indicating that the model is sufficient to navigate the design space. The developed models are further illustrated with the response surface and its respective contour plots in Figure 15, Figure 16, Figure 17 and Figure 18, and the optimum region of the biocarbon formulation is determined. Supplementary information, containing Figures S1–S6, shows residual vs. predicted values, residuals vs. pyrolysis temperature, residuals vs. particle size, residuals vs. filler loading of studentized residuals, and predicted vs. actual data, respectively of the water absorption of the hemp biocarbon-filled hemp fiber-reinforced bio-epoxy composites. Similarly, Supplementary Information, containing Figures S7–S12, shows normal probability plot, residual vs. predicted values, residuals vs. pyrolysis temperature, residuals vs. particle size, residuals vs. filler loading of studentized residuals, and predicted vs. actual data, respectively of the water absorption of the switchgrass biocarbon-filled hemp fiber-reinforced bio-epoxy composites.

#### 2.5.4. Response Surfaces and Contour Plots of the Biocarbon-Filled Hemp Reinforced Biopolymer Composites

**Figure 19:**
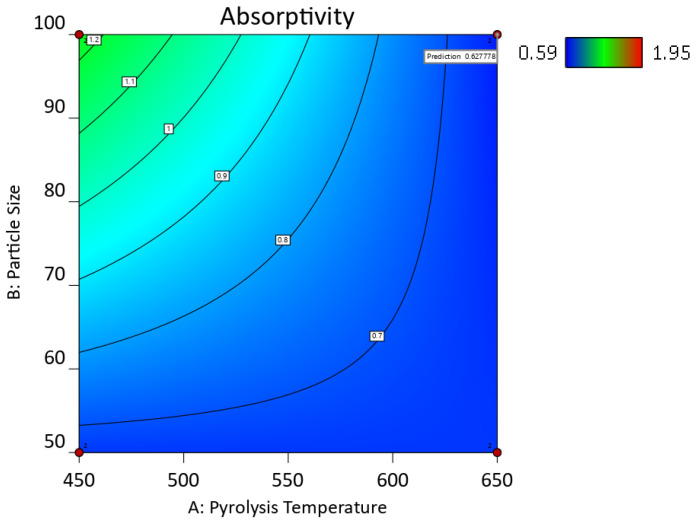
Contour plot of the desirability of 0.972 with the predictability of 0.697 for the water absorptivity of switchgrass biocarbon-filled hemp-reinforced polymer composites when the filler loading is 20%.

The numerical and graphical analysis followed the model development with regression analysis and its adequacy check. Finally, the optimization of the sample design was performed. The objective of this study was to achieve the optimum water absorptivity of the hemp-reinforced biopolymer composites filled with biocarbon fillers. The 3D response surfaces of the water absorptivity with 20% filler loading are shown in Figure 15 and Figure 17, and their 2D contour plots are shown in Figure 16, Figure 18 and Figure 19.

#### 2.5.5. Optimization of the Hemp Composites for the Water Absorptivity

From the contour plots and the 3D surface plots, the conditions for minimum water absorptivity in the composite samples with biofillers were determined. The minimum water absorptivity in the composite samples with biofillers was found at the following conditions: pyrolysis temperature = 650 °C, particle size = 50 µm, and filler loading = 20% from the contour plot shown in Figure 16 and Figure 18. The 2D and 3D desirability plots (Figure S15) and the cube plots (Figures S13 and S14) showing the factors and the predicted values at the optimized conditions are provided in the Supplementary Data.

## 3. Materials and Methods

### 3.1. Biocarbon and Biocomposite Samples Preparation

Altair hemp stalk (*Cannabis sativa* L.) was provided by the Utopia Hemp company, Utopia, ON, Canada, and switchgrass was collected from the OBPC Farmers, ON, Canada. Biocarbon was produced through in-house pyrolysis of the hemp stalk and switchgrass feedstock. Hemp stalk and switchgrass were ground and sieved to 200 microns. Biocarbon was obtained by pyrolyzing the hemp and switchgrass feedstock at 3 different temperatures (450, 550, and 650 °C) in a nitrogen environment. The nitrogen flow was set to 0.75 L/min, the heating rate was kept at 10 °C/min, and the residence time was 30 min. The biocarbon was left to cool under nitrogen conditions inside the reactor.

**Figure 20:**
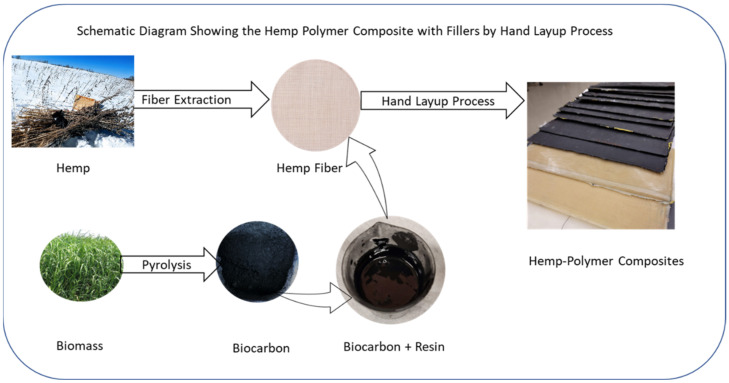
Composite sample preparation by hand-layup technique.

Ecopoxy Biopoxy 36 resin with hardener was purchased from Kitchener Fiberglass, Kitchener, ON, Canada. The biocarbon was crushed, and particle sizes below 50 µm, 75 µm, and 100 µm were obtained with the help of the V8SH 50U, 8X2 316L 200 mesh, and V8SH 50U, respectively, on the sieve shaker AS 200. Resin and hardener were taken in the ratio of 4:1 by volume. Six plies of rectangular hemp fabric pieces were used in each sample. The total weight of the fabric in each sample was 46.6 (±1.22) g. The biocarbon filler was added to the resin at 10%, 15%, and 20% by resin weight. Then, the resin–hardener–biocarbon solution was stirred for 2 min. Finally, the composite samples were prepared by implementing the hand-layup technique. The composite was left to cure under vacuum (55 kPa) for 24 h before their extraction for analyses. The schematic of the sample preparation has been presented in Figure 20.

### 3.2. Physiochemical Analyses

The proximate analysis of the raw samples (hemp stalk and switchgrass) and their biochar samples at various temperatures were performed as per the ASTM standard. ASTM D3173 [62] was followed to calculate the moisture in the samples. D3175-20 [63] was followed to analyze the volatile matter in the samples. ASTM standard E1755-01 [64] was adhered to find the ash in the samples. The fixed carbon was calculated from the difference.

Flash 2000 Organic Elemental Analyzer CHNS-O Analyzer (Thermo-Fisher Scientific, Waltham, MA, USA) was used to perform the ultimate analysis of the samples. Carbon, oxygen, nitrogen, and sulfur were determined by the instrument. The oxygen content was determined from the difference (by subtracting the C, H, N, S, and ash determined from the proximate analysis).

### 3.3. Water Absorption Behaviour

The water absorption test was performed following the ASTM standard D570-98 (2018) [65]. The samples were prepared by heating them at 50 °C, cooling them in a desiccator, and immediately weighing them to the nearest 0.001 g. The rectangular test coupons were taken in the size of 76.2 mm × 25.4 mm. The long-term immersion method was implemented where the conditioned specimens were placed in the deionized water maintained at 23 °C. The samples were removed from the water one at a time, wiped off with a cloth, and weighed. The weight measurements were taken for 5 weeks of immersion. The surface water was wiped off with a dry cloth and weighed immediately to the nearest 0.001 g. When the increase in weight per 2-week period (shown by 3 consecutive weightings) averaged less than 1% of the total increase in weight or 5 mg (whichever is greater), the weighing was stopped. At this point, the samples were considered to have been substantially saturated. The water absorption percentage was calculated as [58,66]:

WA (%) = (W_t_ − W_0_)/W_0_ × 100%,

where WA (%) is the percentage of water absorbed by the samples as compared to the dead weight, W_t_ is the weight of water absorbed sample at time t, and W_0_ is the weight of the dry sample.

### 3.4. Tensile Properties

The mechanical properties of the composite specimens of the samples were studied. The tensile properties of the samples were measured before and after the samples were fully saturated in water. Instron 5969 was used to perform the tensile tests. The grip-to-grip separation was maintained at 100 mm for all samples. Each sample was tested twice. A 50 kN cell was implemented, and no extensometer was used during the test. The pulling rate was kept at 5 mm/min until the samples broke apart. Tensile strength, tenacity, maximum load, energy at the break, and elongation at the break were recorded. The flexural properties of the samples were determined before and after the water absorption tests with Instron 5965.

### 3.5. Flexural Test

A 3-point bending (flexural test) was performed with Instron 5965 with 5 kN cell as per the ASTM D790-17 [67]. The samples were sized as per the standard, and the tests were repeated for accuracy. The rate of the load was taken as per the following formula:

R = Z × L2/6D

where R = crosshead speed (mm/min), Z = 0.01, L = distance between the supports = 50 mm, and D = Depth of the sample (mm). Hence, R = 4.2/D.

### 3.6. Composites’ Swelling Behaviour

The thickness of the samples was measured before performing the immersion test. The samples’ thickness after the water immersion test was again recorded when the samples were fully saturated. Three measurements were taken for each sample. The increase in thickness of the composite samples gives us the swelling nature of the composite samples due to the water absorption.

Mathematically,

S_w_ = (T_s_ − T_o_)/T_o_ × 100%

where S_w_ is the swelling percentage of the samples, T_s_ is the sample thickness at saturation point, and T_o_ is the sample thickness before the water absorption test.

### 3.7. Fourier Transform IR

FTIR analyses of the biocarbon samples were performed on Nicolet 6700 FT-IR (Thermo Electron Corporation, Waltham, MA, USA). Each sample was analyzed twice. The Nicolet 6700 FTIR spectrometer is a powerful tool for analyzing the composition of materials using Fourier Transform Infrared (FTIR) spectroscopy. The crushed sample is positioned in the infrared beam, and 20 scans were performed in the spectral range of 650 to 4000 cm^−1^ with sensitivity of 75 cm^−1^. The generated interferogram of signal as a function of time is Fourier transformed to obtain the spectrum. The resulting spectrum was analyzed by identifying the peaks corresponding to specific chemical bonds and functional groups and comparing them to the library of spectra to identify unknown compounds.

### 3.8. Scanning Electron Microscopy

The surface morphology of the composites was studied under the FEI Quanta 250 Field Emission Scanning Electron Microscope (FE-SEM) by generating the magnified cross-sectional views on the tensile-tested samples. The accelerating voltage was set to 20 kV, and a working distance of 10 mm was maintained. The instrument was set to 4.19 × 10^−6^ bar vacuum pressure. The samples obtained from the tensile tests were fractured in normal room conditions by shearing with a bolt cutter. Before their microscopic imaging, each sample was sputter-coated under Helium in a Desk V Denton Vacuum instrument.

### 3.9. Experimental Design and Statistical Analysis

**Figure 21:**
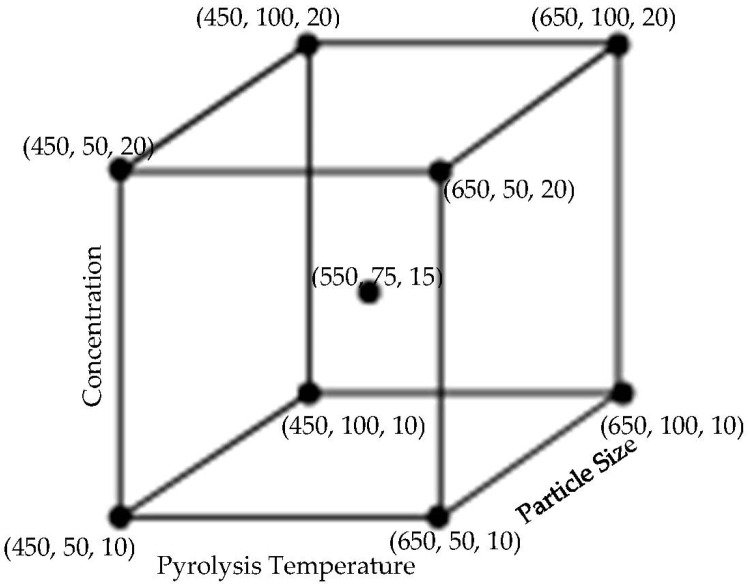
Schematic diagram of the Design of Experiments where the nodes represent the biocarbon characteristics in the order of (temperature in °C, particle size in microns, and particle concentration in percentage).

The pyrolysis temperatures were selected based on past research work. The biocarbon size and the amount were also chosen based on experience, the amount being not more than 20%, and the size not exceeding 100 microns. A 2^3^ full factorial design of experiments with repeated central points to study the curvature of the response function was performed using the Design-Expert 12 (Stat-Ease Inc., Minneapolis, MN, USA). The effects of two levels of three variable factors: pyrolysis temperature, biocarbon concentration, and biocarbon size on the measured water absorptivity were analyzed. The schematic of the design is presented in Figure 21. Response surface methodology was used to perform the statistical optimization of the level of the chosen variable factors to obtain the minimum water absorption.

Furthermore, numerical optimization of the process condition, biocarbon percentage, and size based on desirability were performed. The response measured was the water absorptivity of the composite samples. The lack-of-fit and error components were used to determine these factors’ significance and the model’s desirability.

## 4. Conclusions

The interaction effect of the design parameters (pyrolysis temperature, particle size, and the filler loading of the biocarbon particle fillers) on the water absorption of the hemp-reinforced biopolymer composites has been studied. The incorporation of biocarbon fillers in the hemp biopolymer composites reduces the average water absorptivity by 44.17%. Similarly, the water diffusivity reduces by 42.02% when biocarbon is added to the hemp-biopolymer composites. The unfilled composites show a higher rate of water diffusion and water absorption and hence, earlier water saturation. The unfilled unreinforced biopolymer composites with the least water absorption and negligible swelling offer poor mechanical strength. Regardless of the type of biomass feedstock, the least water absorptivity was achieved with the biocarbon fillers at the following optimized conditions: pyrolysis temperature: 650 °C, biocarbon loading: 20%, and biocarbon particle size: 100 µm. The water absorptivity of hemp biocarbon-filled hemp composites and switchgrass biocarbon-filled hemp composites at the optimum conditions was 0.72 × 10^−6^ g/m^2^.s^1/2^ and 0.73 × 10^−6^ g/m^2^.s^1/2^, respectively, which is lower by more than 70% from that of the unfilled hemp biopolymer composites. The biocarbon at 650 °C showed the least thickness swelling of the composite samples; biocarbon fillers at higher temperatures caused lesser thickness swelling due to water because of its higher porosity and lower surface area, which reduces its ability to absorb water and swell. Biocarbon-filled hemp composites showed higher flexural strength and energy at the break than the hemp composites without fillers due to the enhanced mechanical interlocking between the filler particles and the matrix materials, resulting in increased interfacial strength and improved load transfer between the components of the biocomposite materials. The water absorbed samples demonstrated increased energy at the break while significantly compromising their flexural properties. This increased energy at break was due to the moisture acting as a plasticizer; water causing the microcracking and hydrolysis. The smaller the filler particle size, the lower the composite’s water diffusivity, whereas the higher particle size of the biocarbon fillers in composites minimizes the water absorption because it creates a more compact and dense structure, which reduces the amount of void space available for water to penetrate the composite material. Additionally, the higher the biocarbon loading, the poorer the composite’s tensile energy at the break of the hemp-reinforced composite samples due to the filler agglomeration, reduced polymer–filler interactions, reduced polymer chain mobility, and inadequate dispersion of the filler.

## Figures and Tables

**Table 1:** Water Absorption Property of Composite Samples with Various Formulations.

Factor 1	Factor 2	Factor 3	Switchgrass Biocarbon	Hemp Biocarbon
A: Pyrolysis Temperature	B: Particle Size	C: Filler Loading	Water Absorptivity	Water Absorptivity
650	100	10	0.97	1.52
650	100	20	0.76	1.49
650	50	20	0.59	0.87
650	50	10	1.95	1.36
650	50	20	0.64	1.1
450	100	20	1.07	1.22
550	75	15	1.59	1.77
450	100	20	1.23	1.93
650	100	10	1.2	1.49
450	50	10	1.69	1.75
450	100	10	1.83	1.93
650	50	10	1.53	1.4
450	100	10	1.92	2.04
450	50	20	0.72	1.17
450	50	20	0.6	1.7
450	50	10	1.7	1.7
550	75	15	0.97	1.46
650	100	20	0.69	1.4

**Table 2:** Full-Model ANOVA Table for the Water Absorptivity as Response of the Biocarbon-Filled Hemp-Reinforced Polymer Composites.

Hemp Biocarbon-Filled Composites						Switchgrass Biocarbon-Filled Composites				
Source	SS	df	MS	F-Value	*p*-Value	SS	df	MS	F-Value	*p*-Value
Model	1.530	7	0.219	5.310	0.012	3.730	7	0.533	14.350	0.000
A-Pyrolysis Temperature	0.258	1	0.258	6.260	0.034	0.369	1	0.369	9.930	0.012
B-Particle Size	0.473	1	0.473	11.480	0.008	0.004	1	0.004	0.105	0.753
C-Filler Loading	0.597	1	0.597	14.500	0.004	2.630	1	2.630	70.800	0.000
AB	0.011	1	0.011	0.255	0.626	0.369	1	0.369	9.930	0.012
AC	0.101	1	0.101	2.450	0.152	0.019	1	0.019	0.509	0.494
BC	0.092	1	0.092	2.220	0.170	0.289	1	0.289	7.770	0.021
ABC	0.001	1	0.001	0.026	0.876	0.052	1	0.052	1.390	0.268
Curvature	0.045	1	0.045	1.100	0.322	0.013	1	0.013	0.361	0.563
Pure Error	0.370	9	0.041			0.335	9	0.037		
Total	1.950	17				4.080	17			

**Table 3:** Fit Statistics (the predicted R^2^ against the adjusted R^2^) for the Full Model of biocarbon-filled hemp reinforced polymer composites.

Samples	Std. Dev.	Mean	C.V. %	R^2^	Adjusted R^2^	Predicted R^2^
Hemp biocarbon-filled composites	0.2029	1.47	13.77	0.8052	0.6537	0.2207
Switchgrass biocarbon-filled composites	0.1928	1.2	16.03	0.9178	0.8538	0.671

**Table 4:** ANOVA table for the Reduced Model for the Water Absorptivity as a Response of the Biocarbon-Filled Hemp Composites.

Hemp Biocarbon-Filled Composites						Switchgrass Biocarbon-Filled Composites				
Source	SS	df	MS	F-Value	*p*-Value	SS	df	MS	F-Value	*p*-Value
Model	1.330	3	0.442	10.000	0.001	3.660	4	0.915	28.140	<0.0001
A-Pyrolysis Temperature	0.258	1	0.258	5.820	0.030	0.369	1	0.369	11.350	0.005
B-Particle Size	0.473	1	0.473	10.680	0.006					
C-Filler loading	0.597	1	0.597	13.490	0.003	2.630	1	2.630	80.970	<0.0001
AB						0.369	1	0.369	11.350	0.005
BC						0.289	1	0.289	8.890	0.0106
Residual	0.619	14	0.044			0.423	13	0.033		
Lack of Fit	0.249	5	0.050	1.210	0.378	0.088	4	0.022	0.592	0.6775
Pure Error	0.370	9	0.041			0.335	9	0.037		
Total	1.950	17				4.080	17			

**Table 5:** Fit Statistics (the predicted R^2^ against the adjusted R^2^) for the Reduced Model of the Biocarbon-Filled Hemp Composite Samples.

Samples	SD	Mean	C.V. %	R^2^	Adjusted R^2^	Predicted R^2^	PRESS
Hemp biocarbon composites	0.2103	1.47	14.28	0.6818	0.6136	0.4728	1.03
Switchgrass biocarbon composites	0.1803	1.2	14.99	0.8965	0.8646	0.8329	0.68

## Data Availability

No new data were created.

## Supplementary Materials

The following supporting information can be downloaded at: https://www.mdpi.com/article/10.3390/ijms24076093/s1.
