# Supplementary material for: The Interaction Effect of the Design Parameters on the Water Absorption of the Hemp-Reinforced Biocarbon-Filled Bio-Epoxy Composites
Source: Int J Mol Sci. 2023 Mar 23;24(7):6093. doi: 10.3390/ijms24076093 (PMC10094122; doi:10.3390/ijms24076093)
Supplement: Supplementary file 1 [file ijms-24-06093-s001.zip › ijms-2206154-supplementary.pdf]

Article

The Interaction Effect of the Design Parameters on the Water Absorption of the Hemp-Reinforced Biocarbon-Filled Bio-Epoxy Composites

Raj Kumar Dahal [1], Bishnu Acharya [2,*] and Animesh Dutta [1,*]

[1] Bio-Renewable Innovation Lab, School of Engineering, University of Guelph, Guelph, ON N1G 2W1, Canada
[2] Department of Chemical and Biological Engineering, University of Saskatchewan, Saskatoon, SK S7N 5A9, Canada
* Correspondence: bishnu.acharya@usask.ca (B.A.); adutta@uoguelph.ca (A.D.)

SUPPLEMENTARY DATA

Table S1: The chemical and proximate analyses of the biomasses, and their biocarbon products at 450 °C, 550 °C, and 650 °C.

In (%)	H	H450	H550	H650	S	S450	S550	S650
Moisture	7.26 (±0.03)	3.17 (±0.01)	1.56 (±0.11)	1.59 (±0.09)	5.47 (±0.09)	1.19 (±0.10)	1.28 (±0.20)	0.25 (±0.26)
VM (wet)	73.34 (±0.97)	23.30 (±0.34)	18.94 (±0.57)	14.86 (±0.26)	82.71 (±0.48)	21.52 (±0.02)	11.71 (±0.67)	10.17 (±0.24)
Ash (dry)	3.56 (±0.20)	8.29 (±0.36)	13.05 (±0.24)	13.88 (±0.19)	1.35 (±0.14)	10.86 (±0.26)	12.47 (±0.03)	8.22 (±0.27)
FC (dry)	8.53	62.08	66.45	68.08	5.01	65.23	73.26	81.11
N	0.35 (±0.05)	0.82 (±0.00)	0.85 (±0.05)	0.71 (±0.08)	1.41 (±0.08)	1.40 (±0.14	1.10 (±0.07)	0.98 (±0.03)
C	45.34 (±0.25)	71.06 (±0.26)	73.88 (±0.52)	73.54 (±0.21)	43.35 (±0.27)	73.20 (±0.02)	80.54 (±0.25)	82.03 (±0.39)
H	5.91 (±0.15)	2.90 (±0.03)	2.16 (±0.01)	1.67 (±0.07)	5.93 (±0.09)	2.89 (±0.18)	2.35 (±0.05)	1.62 (±0.01)
S	0 (±0.00)	0.03 (±0.04)	0 (±0.00)	0.05 (±0.01)	0.09 (±0.00)	0 (±0.00)	0 (±0.00)	0 (±0.00)
O	47.05	14.32	10.64	15.81	45.66	14.22	2.95	1.48

FC= Fixed Carbon, VM= Volatile Matter, H=Hemp stalk, S=Switchgrass, H450= Biocarbon from hemp at 450 °C, H550=H Biocarbon from hemp at 550 °C, H650=Biocarbon from hemp at 650 °C, S450 = Biocarbon from switchgrass at 450 °C, S550= Biocarbon from switchgrass at 550 °C, S650= Biocarbon from switchgrass at 650 °C.

Table S2: Showing the average absorption coefficient and their respective water diffusion rate of hemp composite with biocarbon fillers.

Hemp Biocarbon Composite Samples	Average Absorptivity ($x10^{-6}$ (g/m^2. s$^{1/2}$)	Average Diffusivity ($x10^{-5}$ mm^2/s)	Switchgrass Biocarbon Composite Samples	Average Absorptivity ($x10^{-6}$ (g/m^2. s$^{1/2}$)	Average Diffusivity ($x10^{-5}$ mm^2/s)
H45-50-10	1.80 (±0.244) a	6.74 (±1.178) A	S45-50-10	1.72 (±0.031) abc	5.17 (±0.332) BC
H45-50-20	1.04 (±0.129) bcd	2.16 (±0.369) CD	S45-50-20	0.80 (±0.162) d	2.14 (±0.431) D
H45-100-10	1.99 (±0.054) a	7.69 (±0.277) A	S45-100-10	1.74 (±0.150) abc	6.40 (±0.842) ABC
H45-100-20	0.96 (±0.188) cd	2.54 (±0.897) CD	S45-100-20	1.15 (±0.084) cd	4.35 (±0.291) BCD
H55-75	1.62 (±0.152) ab	6.15 (±0.841) AB	S55-75	1.28 (±0.308) bcd	3.60 (±1.011) CD
H65-50-10	1.38 (±0.023) abcd	3.46 (±0.180) BCD	S65-50-10	1.98 (±0.294) a	7.08 (±1.661) AB
H65-50-20	0.98 (±0.115) bcd	2.55 (±0.932) BCD	S65-50-20	0.75 (±0.145) d	1.72 (±0.493) D
H65-100-10	1.47 (±0.052) abc	5.14 (±0.533) ABC	S65-100-10	2.00 (±0.222) ab	9.05 (±0.676) A
H65-100-20	0.72 (±0.007) d	1.82 (±0.139) D	S65-100-20	0.84 (±0.118) d	2.29 (±0.157) D

Table S3: Showing the average absorption coefficient and their respective water diffusion rate of composites with and without reinforced hemp fiber and biocarbon filler.

Samples	Average Absorptivity ($x10^{-6}$ (g/m^2. s$^{1/2}$)	Average Diffusivity ($x10^{-5}$ mm^2/s)	Average Swelling (mm/mm) %
HE	2.41 (±0.033)	7.67 (±0.139)	12.92 (±0.251)
HaR	0.17 (±0.004)	0.02 (±0.000)	0.39 (±0.023)

Table S4: Showing the average swelling of the biocomposite samples.

Sample Code	Pyrolysis Temperature (°C)	Particle Size (µm)	Filler Loading (wt.%)	Hemp Biocarbon Composite Swelling %	Switchgrass Biocarbon Composite Swelling %
45-50-10	450	50	10	10.43 (±0.28) ab	10.50 (±0.50) AB
45-50-20	450	50	20	7.39 (±0.95) cd	7.44 (±0.32) CD
45-100-10	450	100	10	11.07 (±0.30) a	11.20 (±0.13) A
45-100-20	450	100	20	7.77 (±0.48) bcd	9.01 (±0.42) BC
55-75	550	75	15	9.84 (±0.28) abc	7.53 (±0.71) CD
65-50-10	650	50	10	8.35 (±0.12) bcd	9.92 (±0.62) AB
65-50-20	650	50	20	7.35 (±0.29) cd	5.56 (±0.46) E
65-100-10	650	100	10	8.46 (±1.61) abcd	10.67 (±0.47) AB
65-100-20	650	100	20	6.23 (±0.25) d	6.94 (±0.17) DE

Table S5: Showing the average flexural modulus (MPa) of the hemp-composites before and after the water absorption.

				Flexural Modulus (MPa)			
				Hemp Biocarbon		Switchgrass Biocarbon	
Sample Code	Pyrolysis Temperature (°C)	Particle Size (µm)	Filler Loading (wt.%)	Before	After	Before	After
45-50-10	450	50	10	68.90 (±6.57)	37.85 (±0.535)	76.63 (±0.73)	43.80 (±1.703)
45-50-20	450	50	10	81.65 (±8.72)	40.38 (±8.986)	120.59 (±35.72)	56.72 (±2.031)
45-100-10	450	100	10	61.02 (±2.43)	39.81 (±2.245)	72.01 (±5.43)	36.69 (±2.936)
45-100-20	450	100	20	95.83 (±18.44)	66.96 (±15.858)	119.79 (±21.03)	59.33 (±3.735)
55-75	550	75	15	92.51 (±4.13)	35.04 (±2.224)	100.18 (±5.83)	59.44 (±23.648)
65-50-10	650	50	10	70.57 (±2.51)	33.95 (±2.974)	63.22 (±10.35)	43.05 (±3.330)
65-50-20	650	50	20	89.52 (±3.56)	59.57 (±0.305)	145.37 (±6.40)	74.76 (±6.132)
65-100-10	650	100	10	87.85 (±4.72)	58.69 (±8.085)	101.70 (±8.55)	47.86 (±7.641)
65-100-20	650	100	20	137.25 (±10.23)	97.29 (±14.011)	141.92 (±19.35)	88.40 (±25.570)

Table S6: Showing the average energy at break of the hemp-composites before and after the water absorption.

				Hemp Biocarbon Composites Energy at Break (J/m^2)		Switchgrass Biocarbon Composites Energy at Break (J/m^2)	
**Sample Code**	**Pyrolysis Temperature (°C)**	**Particle Size (μm)**	**Filler Loading (wt.%)**	**Before**	**After**	**Before**	**After**
45-50-10	450	50	10	87.86 (±4.51)	201.09 (±44.78)	83.03 (±19.72)	212.45 (±9.40)
45-50-20	450	50	20	61.39 (±6.84)	176.70 (±3.82)	67.79 (±7.88)	143.65 (±27.36)
45-100-10	450	100	10	113.12 (±7.36)	263.20 (±49.20)	124.94 (±19.89)	236.66 (±8.16)
45-100-20	450	100	20	73.93 (±4.64)	165.06 (±30.70)	78.67 (±16.62)	148.85 (±17.43)
55-75	550	75	15	107.06 (±18.98)	206.68 (±41.29)	96.39 (±22.67)	168.11 (±11.83)
65-50-10	650	50	10	80.03 (±4.83)	206.38 (±35.84)	65.46 (±10.27)	219.22 (±5.15)
65-50-20	650	50	20	72.14 (±3.71)	172.98 (±9.04)	80.94 (±7.35)	149.29 (±46.88)
65-100-10	650	100	10	124.80 (±17.29)	201.93 (±39.48)	83.74 (±2.75)	184.42 (±3.95)
65-100-20	650	100	20	78.31 (±26.75)	168.44 (±12.33)	70.11 (±26.75)	179.56 (±14.00)

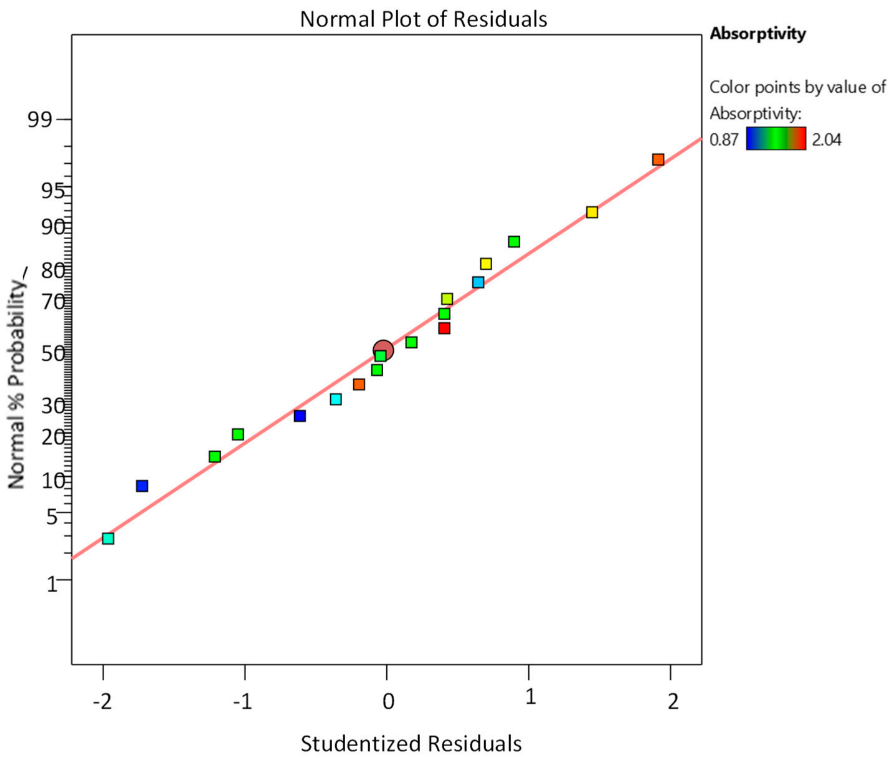

Figure S1: Normal probability plot of Studentized residuals of the hemp biocarbon composite's water absorptivity.

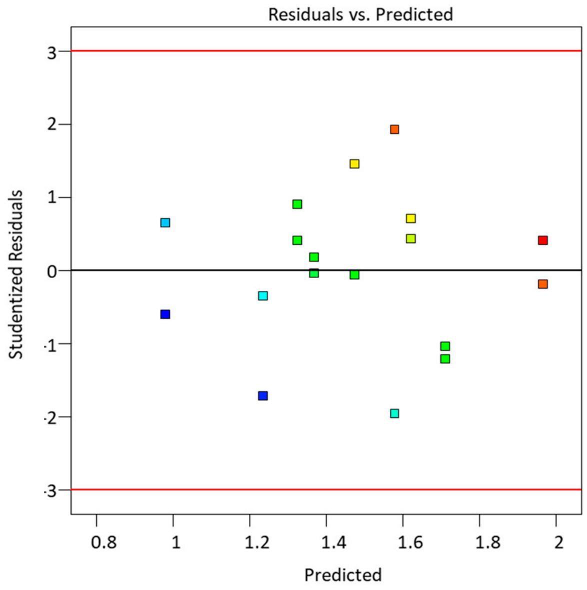

Figure S2: Residuals vs. predicted values of Studentized residuals of the hemp biocarbon composite's water absorptivity.

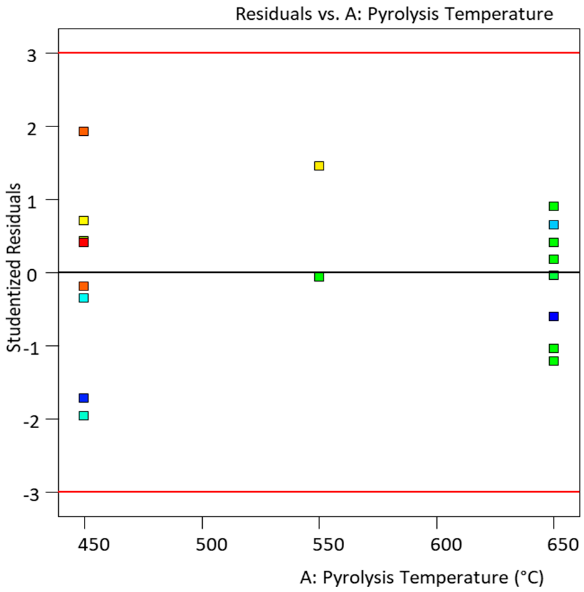

Figure S3: Residuals vs. pyrolysis temperature of Studentized residuals of the hemp biocarbon composite's water absorptivity.

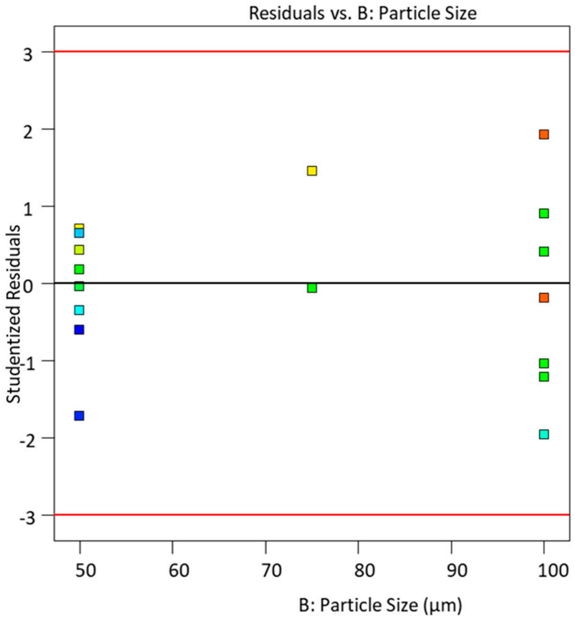

Figure S4: Residuals vs. particle size of Studentized residuals of the hemp biocarbon composite's water absorptivity.

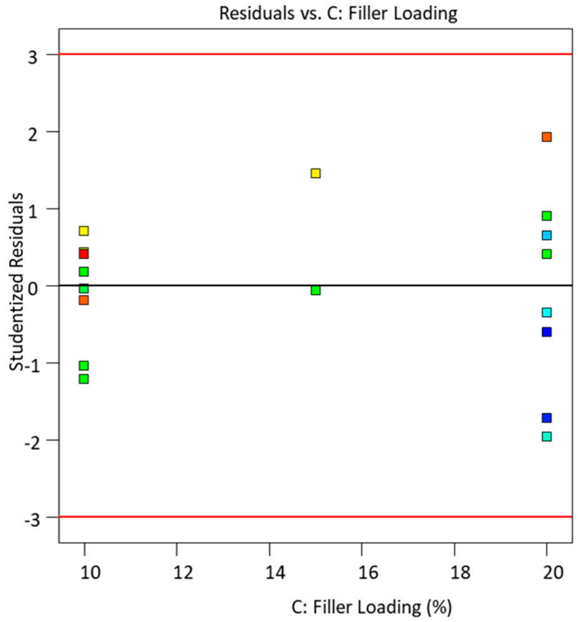

Figure S5: Residuals vs. filler loading values of Studentized residuals of the hemp biocarbon composite's water absorptivity.

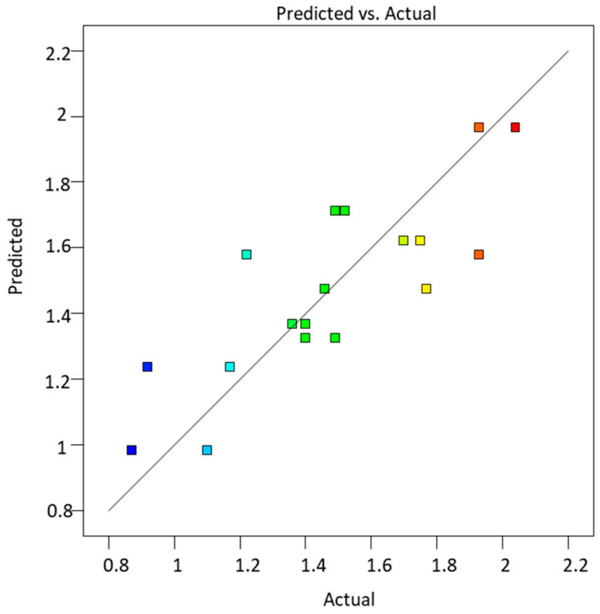

Figure S6: Predicted vs. actual data of the hemp biocarbon composite's water absorptivity.

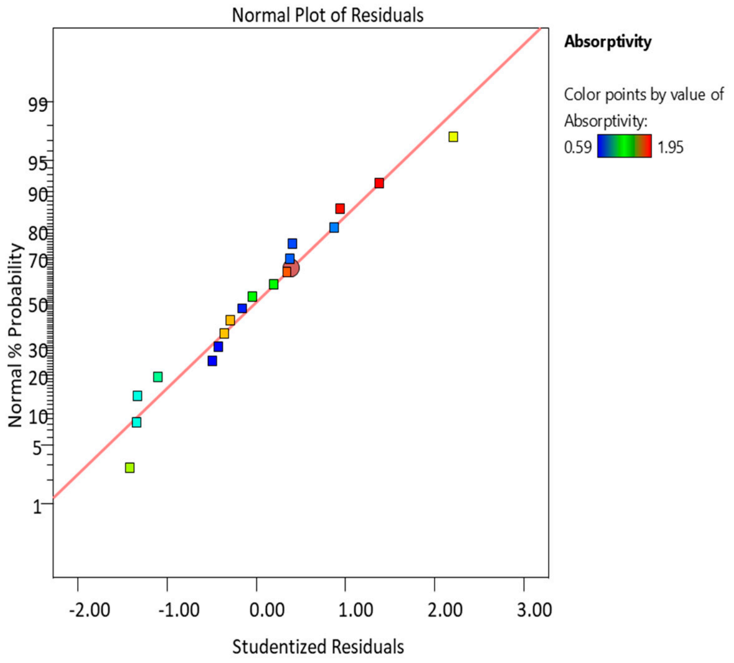

Figure S7: Normal probability plot of Studentized residuals of the switchgrass biocarbon composite's water absorptivity.

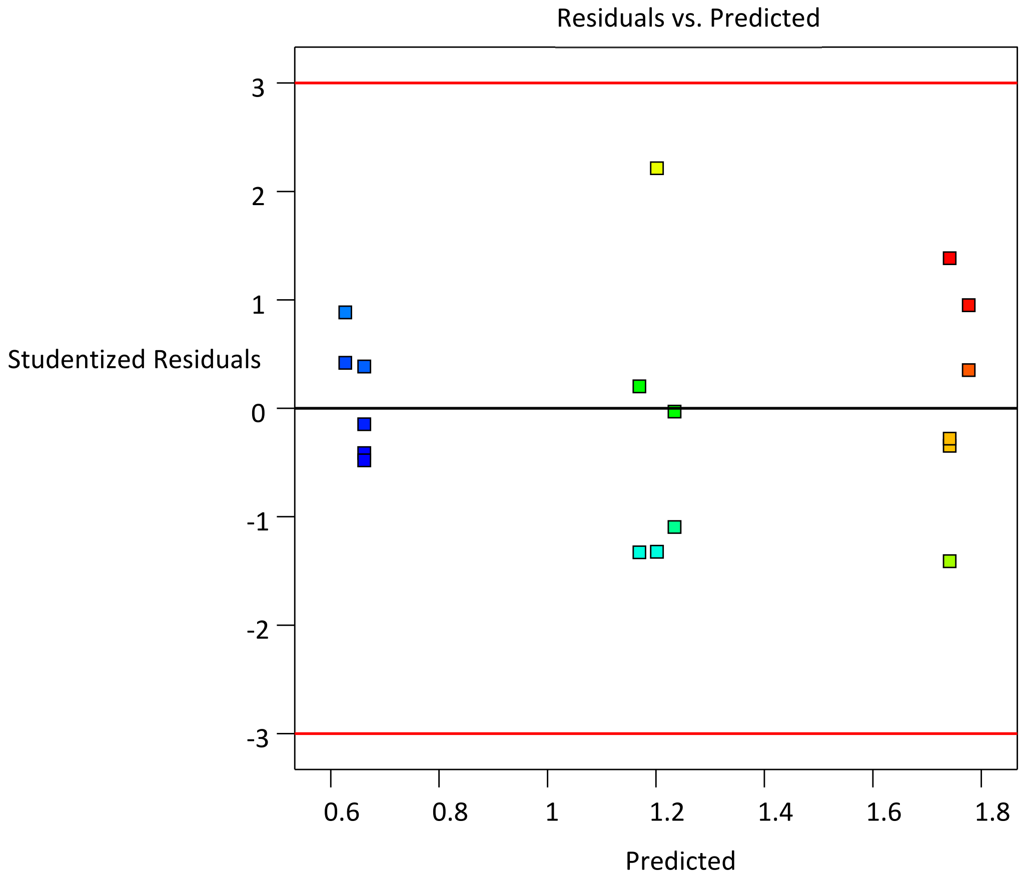

Figure S8: Residuals vs. predicted values plot of Studentized residuals of the switchgrass biocarbon composite's water absorptivity.

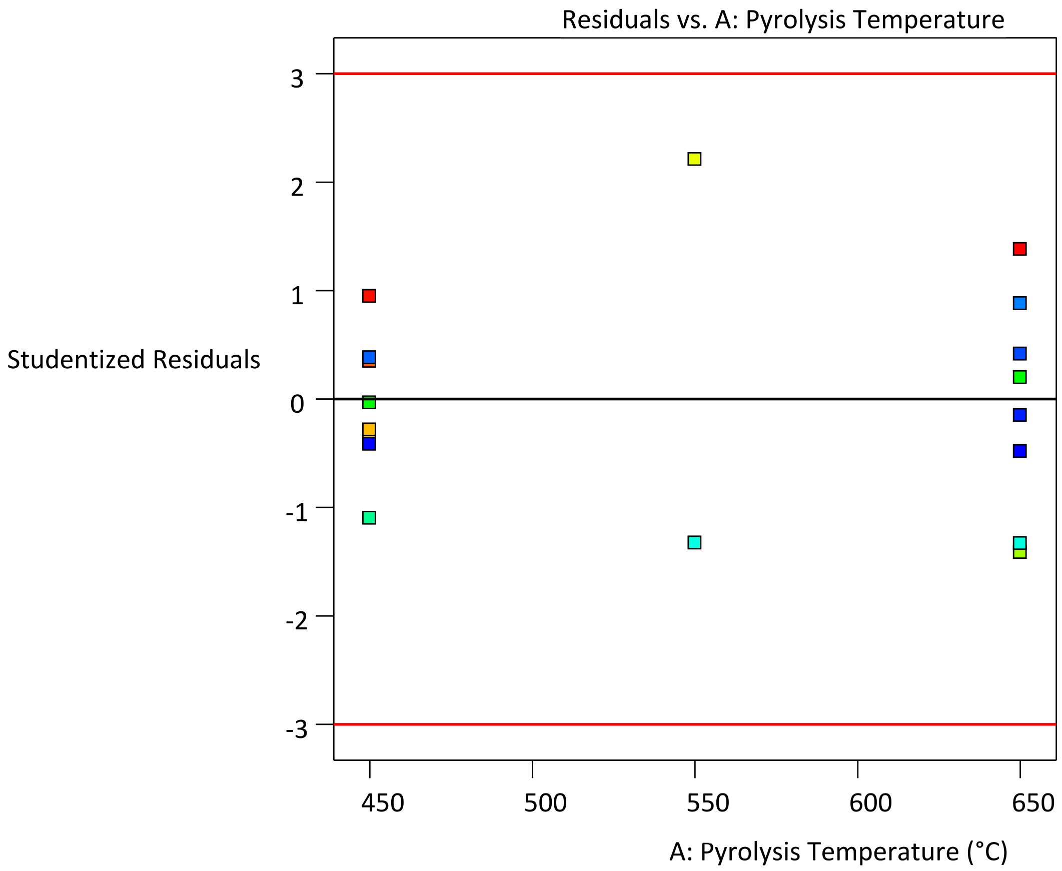

Figure S9: Residuals vs. pyrolysis temperature of Studentized residuals of the switchgrass biocarbon composite's water absorptivity.

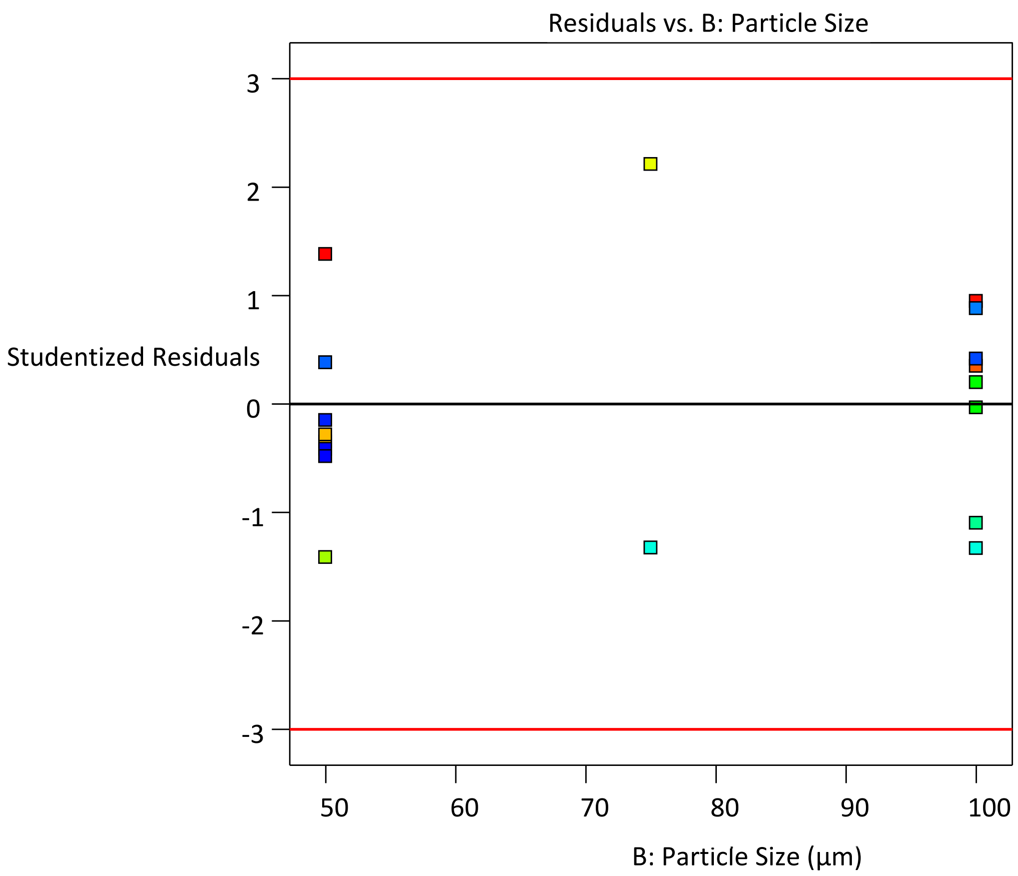

Figure S10: Residuals vs. particle size temperature of Studentized residuals of the switchgrass biocarbon composite's water absorptivity.

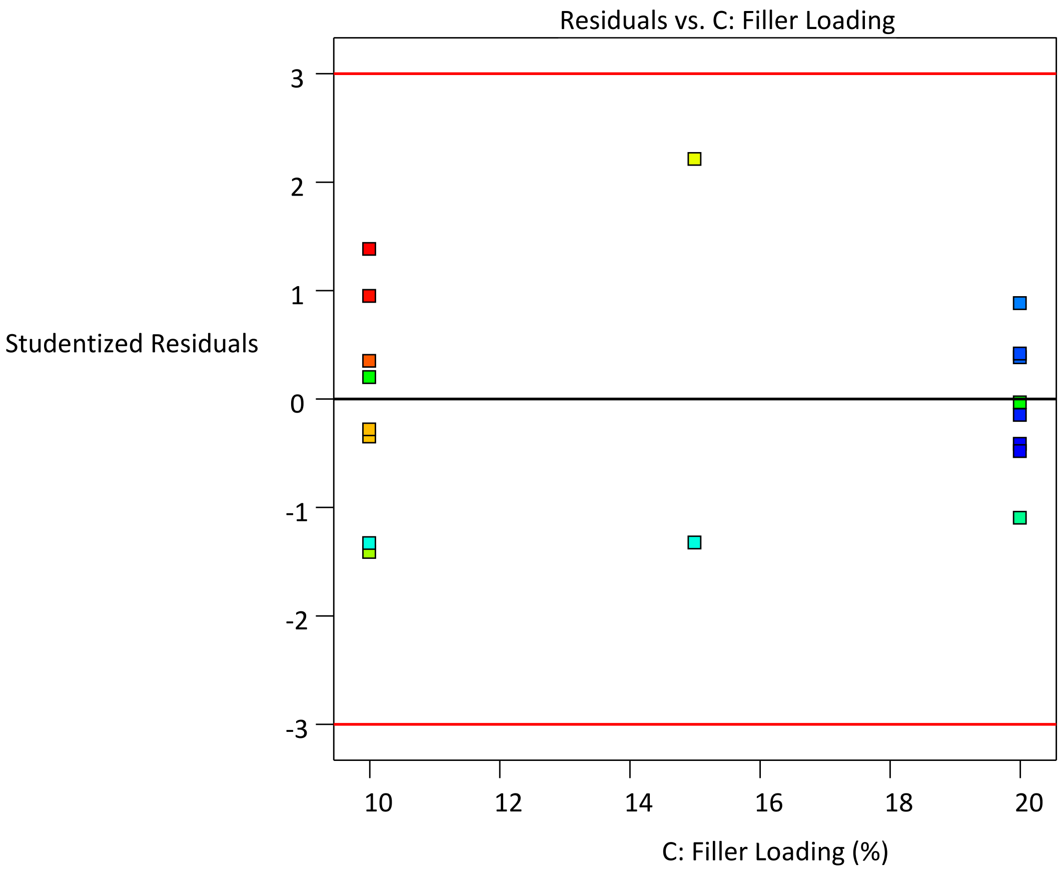

Figure S11: Residuals vs. filler loading of Studentized residuals of the switchgrass biocarbon composite's water absorptivity.

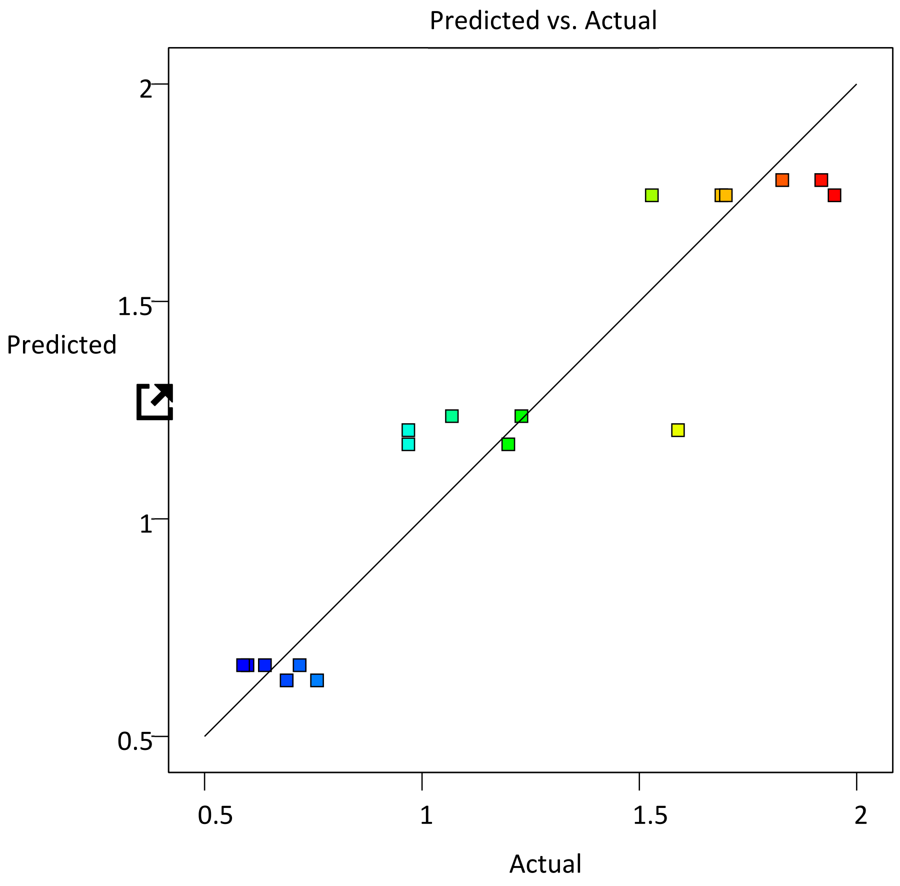

Figure S12: Predicted vs. actual of the switchgrass biocarbon composite's water absorptivity.

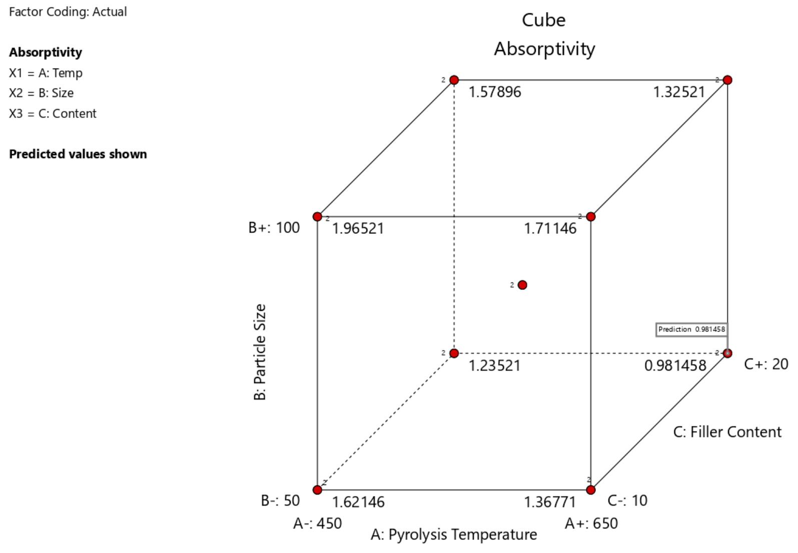

Figure S13: Cube plot showing all factors and the predicted water absorptivity values for the hemp biocarbon-filled hemp-reinforced polymer composites.

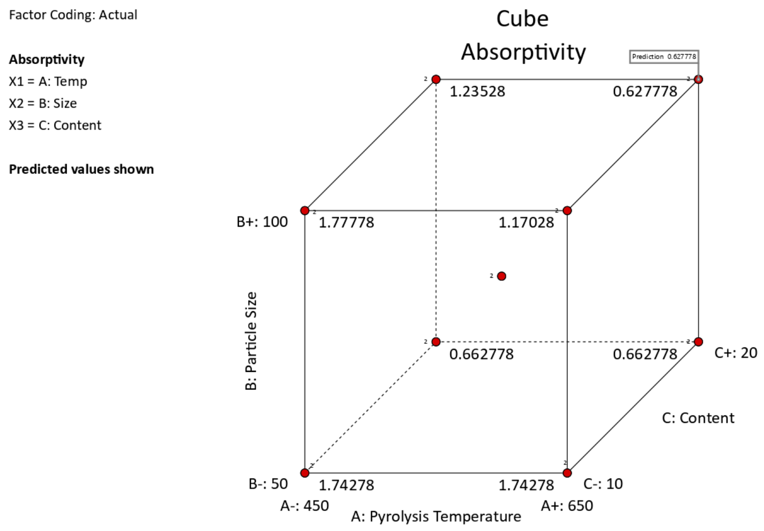

Figure S14: Cube plot showing all factors and the predicted water absorptivity values for the switchgrass biocarbon composites.

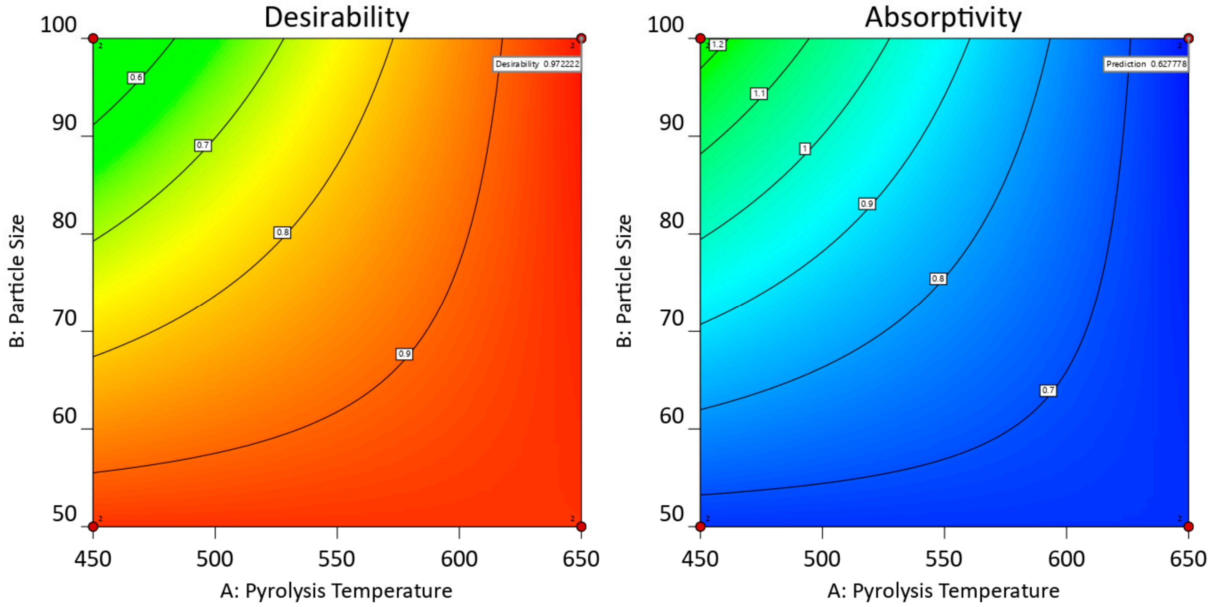

Figure S15: Contour plot showing the desirability of 0.972 with the predictability of 0.697 for the water absorptivity of switchgrass biocarbon-filled hemp-reinforced polymer composites when the filler loading is 20%.

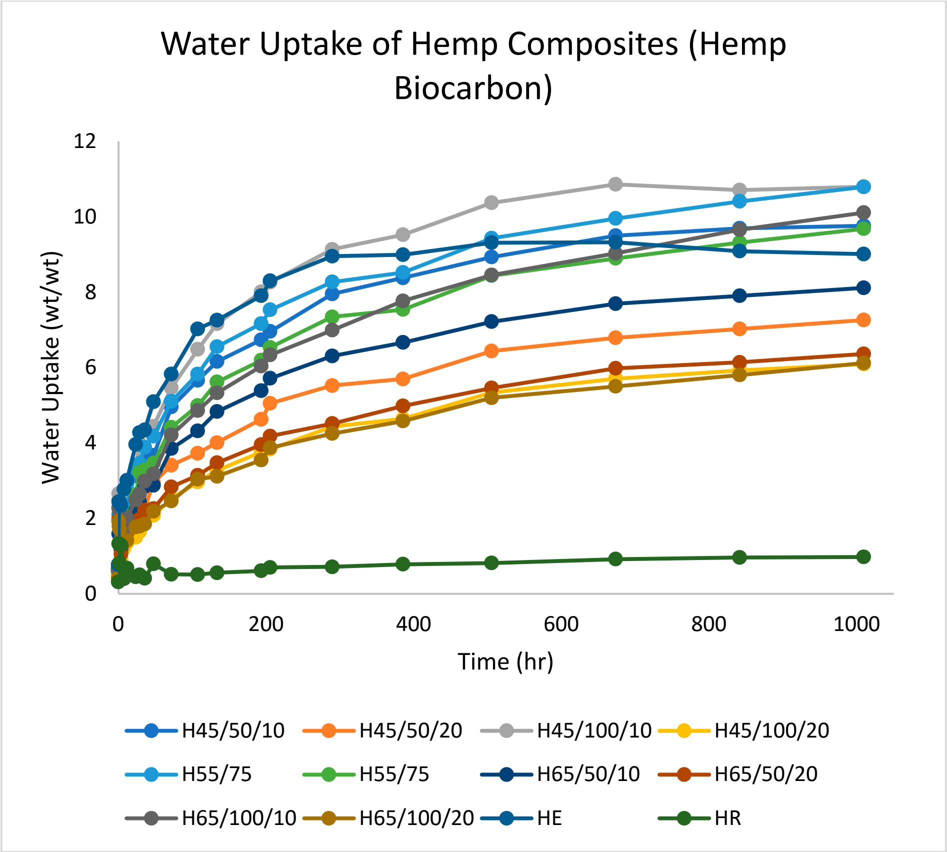

Figure S16: Water absorption curve of hemp composites with hemp biocarbon as a function of time (hr).

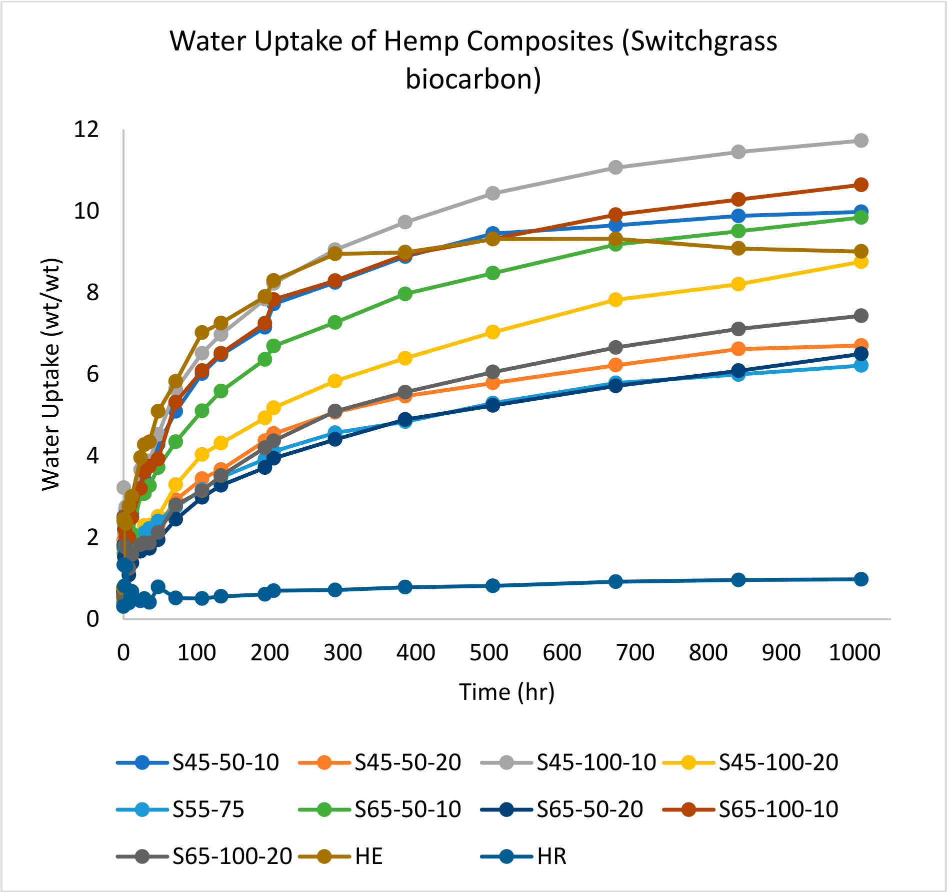

Figure S17: Water absorption curve of hemp composites with switchgrass biocarbon as a function of time (hr).
